# Insights Into the MYB-Related Transcription Factors Involved in Regulating Floral Aroma Synthesis in Sweet Osmanthus

**DOI:** 10.3389/fpls.2022.765213

**Published:** 2022-03-09

**Authors:** Xin Yan, Wenjie Ding, Xiuyi Wu, Lianggui Wang, Xiulian Yang, Yuanzheng Yue

**Affiliations:** ^1^Key Laboratory of Landscape Architecture, Nanjing Forestry University, Nanjing, China; ^2^Co-innovation Center for Sustainable Forestry in Southern China, Nanjing Forestry University, Nanjing, China

**Keywords:** sweet osmanthus, MYB-related, transcription factor, GC-MS, floral aroma

## Abstract

As an important member of the *MYB* transcription factor (TF) family, the *MYB-related* TFs play multiple roles in regulating the synthesis of secondary metabolites and developmental processes, as well as in response to numerous biotic and abiotic stressors in plants. However, little is known regarding their roles in regulating the formation of floral volatile organic compounds (VOCs). In this study, we conducted a genome-wide analysis of MYB-related proteins in sweet osmanthus; 212 *OfMYB-related* TFs were divided into three distinct subgroups based on the phylogenetic analysis. Additionally, we found that the expansion of the *OfMYB-related* genes occurred primarily through segmental duplication events, and purifying selection occurred in all duplicated gene pairs. RNA-seq data revealed that the *OfMYB-related* genes were widely expressed in different organs of sweet osmanthus, and some showed flower organ/development stage-preferential expression patterns. Here, three *OfMYB-related* genes (*OfMYB1R70/114/201*), which were expressed nuclearly in floral organs, were found to be significantly involved in regulating the synthesis of floral VOCs. Only, *OfMYB1R201* had transcriptional activity, thus implying that this gene participates in regulating the expression of VOC synthesis related genes. Remarkably, the transient expression results suggested that *OfMYB1R70*, *OfMYB1R114*, and *OfMYB1R201* are involved in the regulation of VOC synthesis; *OfMYB1R114* and *OfMYB1R70* are involved in accelerating β-ionone formation. In contrast, *OfMYB1R201* decreases the synthesis of β-ionone. Our results deepen our knowledge of the functions of *MYB-related* TFs and provide critical candidate genes for the floral aroma breeding of sweet osmanthus in the future.

## Introduction

*MYB-related* transcription factors (TFs), named for their highly conserved 52 amino acid MYB domain, are predominant and critical members of the MYB TF family in plants (Riechmann and Ratcliffe, 2000; Dubos et al., 2010). MYB TFs can be generally divided into four subfamilies based on the number of MYB domains present: 1R-MYB (MYB-related), R2R3-MYB, 3R(R1R2R3)-MYB, and 4R-MYB proteins (Rosinski and Atchley, 1998). The 1R-MYB are collectively called MYB-related proteins and include proteins with a single or a partial MYB repeat (Dubos et al., 2010). Recently, many studies performing systematic identification and genome-wide characterization of the *MYB* family have reported genes in various plants, such as *Petunia hybrida*, potato (*Solanum tuberosum*), peach (*Amygdalus persica*), and Chinese jujube (*Ziziphus jujuba*) (Zhang et al., 2018; Ji et al., 2019; Liu et al., 2020; Chen et al., 2021). The MYB-related and R2R3-MYB subfamily contain the highest quantity of genes within the MYB family (Liu et al., 2020). To date, R2R3-MYB has been comprehensively characterized and reported to have diverse functions (Nesi et al., 2001; Koes et al., 2005; Ambawat et al., 2013), particularly in enhancing volatile aroma production, promoting anthocyanin accumulation, and responding to abiotic stresses. In contrast, several MYB-related proteins control diverse physiological processes, particularly the regulation of the anthocyanin biosynthesis pathway and the amelioration of multiple abiotic stresses. The first MYB-related TF identified in plants was in potato (*MYBSt1*) (Baranowskij et al., 1994), and since then, the MYB family has gradually gained more attention. However, to date, the function of *MYB-related* genes in regulating the synthesis of floral aroma remains largely unknown.

According to the most recent classification based on the highly conserved motif, MYB-related TFs are divided into three subgroups: the CPC-like subgroup of R3-type single-MYB proteins consisting of CPC (CAPRICE)-like, TRY (TRIPTYCHON)-like, ETC (Enhancer of TRY and CPC)-like and MYBL2; the CCA1-like/R-R subgroup, including the CCA1 (Circadian Clock Associated1)-like, TBP (Telomeric DNA-binding Protein)-like, RAD (RADIALIS)-like, TRF-like, LHY (Late Elongated Hypocotyl), and RVE (Early Phytochrome Responsive1)-like proteins; and the GARP subgroup, which is further classified into the LUX, MYB_CC, KANADI, ARR, HHO, GLK (GOLDEN2-LIKE), and GARP-like subfamilies. The *MYB-related* genes were subsequently confirmed to play fundamental roles as transcriptional regulators, associated repressors, circadian clock, and telomeric repeat-binding proteins in various biological processes. *AtMYBD*, a member of the CCA1-like/R-R subgroup in *Arabidopsis thaliana*, regulates anthocyanin biosynthesis in a circadian clock-related manner (Nguyen and Lee, 2016). *SlTRY*, an R3 MYB member of the CPC-like subgroup, has been confirmed to be involved in anthocyanin biosynthesis and is considered a core regulator of root-hair development and the plant trichome (Rumi et al., 2013). The grape TF AQUILO, a member of the GARP subgroup, increases cold tolerance by promoting the accumulation of raffinose family oligosaccharides (Sun et al., 2018). In recent years, *MYB-related* TFs have been found to be involved in regulating anthocyanin biosynthesis in various pathways and products. The proteins *MdLUX* and *MdPCL*-like, containing a single MYB-like repeat, promote anthocyanin accumulation through DNA hypomethylation in apple (*Malus domestica*) (Li et al., 2021). The functions of MYB-related proteins in metabolite biosynthesis have been characterized in various land plants, for instance, *Arabidopsis*, soybean (*Glycine max*), grapevine (*Vitis vinifera*), lily (*Lilium* spp.), and tiger lily (*Lilium lancifolium*) (Matsui et al., 2008; Niu et al., 2016; Sakai et al., 2019; Yong et al., 2019).

Sweet Osmanthus (*Osmanthus fragrans)* is an evergreen woody flowering member of the Oleaceae. Because of its pleasing floral fragrance and multitudinous flowers, sweet osmanthus is widely cultivated in China as a common landscape tree species (Huang et al., 2015). Sweet osmanthus is relevant to several industries, including tea, food, and perfume production (Li et al., 2020a). The floral fragrance of sweet osmanthus has been comprehensively studied in terms of compound identification and classification. Studies have identified that β-ionone is an essential substance that strongly affects the floral fragrance of sweet osmanthus, and *CCD4* is a vital enzyme regulating the formation of β-ionone (Han et al., 2016). Previous studies have confirmed that *OfCCD4* increases the production of β-ionone, based on the existence of β-carotene, and both *OfWRKY3* and *OfERF61* are positive regulators of the *OfCCD4* gene and enable the synthesis of β-ionone in petals of sweet osmanthus (Han et al., 2016, 2019). According to data on the sweet osmanthus genome, we found that the *OfCCD4* promoter region contains numerous *cis*-acting regulatory elements, thus indicating that *OfCCD4* might be regulated by other TFs (Yang et al., 2018). However, functional identification of related TFs and examination of the transcriptional regulation mechanism of floral volatile organic compounds (VOCs) have been limited. Therefore, studies on the MYB-related TFs’ regulation of enhancing floral VOC production are needed to improve understanding of the mechanism of VOC production and the genetic quality of sweet osmanthus.

Here, we conducted a comprehensive analysis of sweet osmanthus’ MYB-related TF family members, including their potential roles in regulating the formation of floral VOCs. In this study, 212 MYB-related members were identified from the genome of sweet osmanthus, and their chromosomal locations, conserved motifs, and expression patterns were analyzed and visualized. Additionally, we selected three candidate *MYB-related* genes that had flower organ/development stage-preferential expression patterns and then performed detailed gas chromatography-mass spectrometry (GC-MS) analysis of floral VOCs between transient expression plants and empty vector plants.

## Materials and Methods

### Retrieval of Putative OfMYB-Related Family Genes

Sweet osmanthus genomic sequences were acquired from the genome database for *O. fragrans*. The sequence of the MYB DNA binding domain (DBD) was downloaded from the Pfam database (http://pfam.xfam.org/, accession PF00249) (Finn et al., 2016), and HMMER software (version 3.0) with a default *E*-value. The NCBI Batch Web CD-Search Tool (Marchler-Bauer and Bryant, 2004) was further used to assess the numbers of DBDs in the sequences and retain the genes with one DBD. Finally, the putative *MYB-related* genes, according to the number of DBDs and the position information extracted from the General Feature Format (GFF) files, were identified and renamed according to their locations on the chromosomes. The online tool ExPASy was used to verify the physicochemical characteristics of these MYB-related protein sequences, such as the theoretical isoelectric point (pI) and molecular weight.

#### Phylogenetic Analysis and Classification of OfMYB-Related Proteins

A total of 132 AtMYB-related protein sequences^1^ and 212 putative OfMYB-related proteins were selected to construct a phylogenetic tree through neighbor-joining method with a bootstrap score of 1,000 and p-distance parameter (MEGA7.0). The complete amino acid sequences of OfMYB-related proteins were used to obtain the DBDs and validated in the Pfam database. The alignment and visualization of 212 *OfMYB-related* domains and subsequent protein analysis were accomplished with ClustalX.

### Gene Structures and Conserved Motifs of the OfMYB-Related Gene Family

The *OfMYB1R* gene structures and coding sequences (CDSs) were derived from the sweet osmanthus genomic GFF3 file. TBtools software was used to visualize the gene structures, CDS boundaries, and intron distribution (Chen et al., 2020). The MEME Suite webserver was used to screen the conserved motifs in *OfMYB-related* TFs, identifying a maximum of 20 motifs^2^ (Bailey et al., 2009).

### Gene Duplication and Synteny Analysis of OfMYB-Related Genes

The chromosomes corresponding to MYB-related genes were screened and mapped with MG2C.^3^ The Multiple Collinearity Scan toolkit (MCscanX) was used to analyze the tandem and segmental duplication pattern of each *MYB-related* gene.^4^ Ks (synonymous) and Ka (non-synonymous) substitution ratios of gene pairs were evaluated with TBtools software (Chen et al., 2020).

#### Quantitative Real-Time PCR Analysis

Total RNA of sweet osmanthus was extracted from the bud-pedicel (S1), bud-eye (S2), primary blooms (S3), full bloom (S4), and post-bloom flower (S5) stages. Three biological samples were collected and processed with the RNAprep Pure Plant Kit (Tiangen, Beijing, China). After removing the gDNA from the total RNA, cDNA was synthesized with SuperMix (Transgen, Beijing, China) under the following conditions: 42°C for 15 min and 85°C for 5 s (Yue et al., 2021). The qRT-PCR primers for *OfMYB-related* genes were designed in Primer Premier 5.0. The expression of *OfACTIN*, *OfRAN*, and *OfRBP2* genes served as average references for normalization of the expression levels in different flower stages (Zhang et al., 2015) (Supplementary Table 1). Each qRT-PCR assay was performed on three biological samples and three technical replicates. Data of comparative threshold cycle (Ct) values were used to calculate the relative gene expression levels (Yue et al., 2021).

#### Subcellular Localization and Transcriptional Activation Assays

To construct 35S:*OfMYBMYB1R70/114/201*:GFP, the Super 1,300 vector containing the CDS of *OfMYB-relared70/114/201*, without the stop codon was used. *Sma*I and *Kpn*I restriction sites were utilized. *Agrobacterium tumefaciens* (GV3101) containing 35S:*OfMYB1R70/114/201*:GFP and negative control vectors were infiltrated into tobacco (*Nicotiana benthamiana*) leaves for the analysis of subcellular localization. The tobacco plants were grown for 35 days in a growth chamber with 15/9 h light/dark (144 μmol⋅m^–2^⋅s^–1^) and 26 ± 2°C. After 3 days of growth, green fluorescent protein (GFP) fluorescence signals were confirmed in the infiltrated plants *via* DAPI staining with a LSM710 microscope (Zeiss, Germany).

Additionally, through the *Nde*I and *Eco*RI restriction sites, the CDS of *OfMYB-relared70/114/201*, lacking the termination codon, were transformed into the pGBKT7 vector. Then the *Saccharomyces cerevisiae* yeast strain AH109 (WeidiBio, Shanghai, China) was used to obtain the four vectors (three types of pGBKT7-*OfMYB1Rs* and one negative control). Finally, transformed yeast with overexpression vectors were grown in selective defined SD/-Trp, SD/-Trp-Ade, and SD/-Trp-Ade + X-α-gal media with incubation at 30°C for 3 days in the dark.

#### Aroma Compound Analysis of Plants With Transient Expression

To explore the regulatory function of *OfMYB1R70/114/201* genes in volatile metabolic components, we selected tobacco leaves to analyze floral VOCs by transient expression. The method to determine the transient expression of candidate genes in *Nicotiana benthamiana* leaves has been successfully used to explore the functions of genes involved in VOC synthesis, such as *OfTPS1/2* (Zeng et al., 2016), *AtTPS10/14* (Ginglinger et al., 2013), and *MpGPS.SSU* (Yin et al., 2017). GV3101 containing 35S:*OfMYB1R70/114/201*:GFP and empty vectors were used to infiltrate 35-day-old tobacco leaves. Six biological replicates of each OfMYB1R sample were injected after 48 h, and the empty vectors were injected in four biological replicates. After growth for 2 days (light/dark: 15/9 h, 144 μmol⋅m^–2^⋅s^–1^), headspace solid-phase microextraction (SPME), a precise method for measuring aroma, was used to collect the floral VOCs. In every 4-ml SPME vial, 0.4 g samples of fresh leaves were added to the bottom, and 1 μl of 100000-fold diluted ethyl caprate was added to the middle (Shanghai, Macklin Inc., China) (Yang et al., 2018). After exposure at the middle of the capped vials for 35 min at 42°C, the SPME fibers of DB-5 MS were injected into the heated syringe port for 3 min, and desorption was performed at 250°C (Bellefonte, Supelco Inc., United States). Subsequently, the desorbed floral VOCs were identified with the Trace DSQ GC-MS device (Ji, 2021), and the N-alkanes were used to calculate the RI value of the volatile compounds for comparison with the RIL value within DB-5MS (30 × 0.25 × 0.25) to confirm the aroma compound detection^5^ (Ji, 2020, 2021). In the software SIMCA 13.0 (Umetrics AB, Umea, Sweden), divergence in the metabolite levels of plants with transient expression was detected with partial least squares discriminant analysis (PCA-X).

#### Total RNA Extraction and Semi-Quantitative RT-PCR of Plants With Transient Expression

To validate the empty vector and 35S:*OfMYB1R70/114/201*:GFP expression in transgenic tobacco plants, we snap-froze the leaves of 35-day-old tobacco with transient expression 48 h after injection of the vector (light/dark: 15/9 h, 144 μmol⋅m^–2^⋅s^–1^) in liquid nitrogen. According to the RNAprep Pure Plant Kit (Tiangen, Beijing, China) cDNA transcribed from total RNA isolated from the leaves of transgenic plants and empty vector plants was used as the template (Ding et al., 2019). Each *OfMYB1R* assay was performed on six biological samples, and empty vector plants were performed on four biological replicates. The primers of *OfMYB1Rs* for semi-quantitative RT-PCR (RT-PCR) were the same primers used in the qRT-PCR analysis. The gene *NbL25* was used as a reference to normalize the expression levels in tobacco (Supplementary Table 1). The primers for *NbCCD4s* and *NbLCYB*, which are homologs of *OfCCD4* and *OfLCYB*, were selected to verify the participation of candidate genes in regulating floral aroma synthesis (Supplementary Table 1).

## Results

### Identification and Classification of MYB-Related Genes in Sweet Osmanthus

Utilizing the MYB DBD HMM profile (Pfam ID PF00249), MYB and MYB-like domains for the entire sweet osmanthus genome were screened. According to the results, verified by Pfam and NCBI-CDD analysis, a total of 212 non-redundant OfMYB1R protein sequences were identified, a number greater than that for most species (Table 1). Based on the chromosomal position of the MYB-related genes, these genes were successively renamed *OfMYB1R1* to *OfMYB1R212*. Basic information on *OfMYB-related* genes, such as gene ID, renamed name, chromosomal location, pI, and molecular weight, can be found in Supplementary Table 2. The protein sequences are provided in Supplementary Table 3. The total length of the OfMYB-related protein sequences ranged from 82 to 1,816 amino acids, the molecular weights ranged from 9.21 to 199.69 kDa, and the predicted pI values were 4.49–10.1.

**TABLE 1:** Comparison of the *MYB-related* gene family size between *Osmanthus fragrans* and other species.

Type	Species	The number of *MYB-related* genes
Monocots	*Ananas comosus*	65
	*Aegilops tauschii*	97
	*Zoysia japonica*	99
	*Oryza sativa subsp. japonica*	106
	*Eragrostis tef*	143
	*Zea mays*	169
Dicotyledon	*Arachis hypogaea*	44
	*Carica papaya*	51
	*Citrullus lanatus*	54
	*Coffea canephora*	56
	*Vitis vinifera*	57
	*Fragaria vesca*	65
	*Dianthus caryophyllus*	66
	*Cucumis melo*	68
	*Cannabis sativa*	70
	*Capsicum annuum*	76
	*Castanea mollissima*	78
	*Prunus mume*	89
	*Populus euphratica*	91
	*Cajanus cajan*	94
	*Cucumis sativus*	94
	*Ziziphus jujuba*	96
	*Cicer arietinum*	99
	*Citrus sinensis*	117
	*Actinidia chinensis*	138
	*Catharanthus roseus*	144
	*Prunus persica*	145
	*Malus domestica*	146
	*Nicotiana benthamiana*	160
	*Brassica oleracea*	202
	*Osmanthus fragrans*	212
	*Brassica napus*	251
	*Glycine max*	342

**FIGURE 1:**
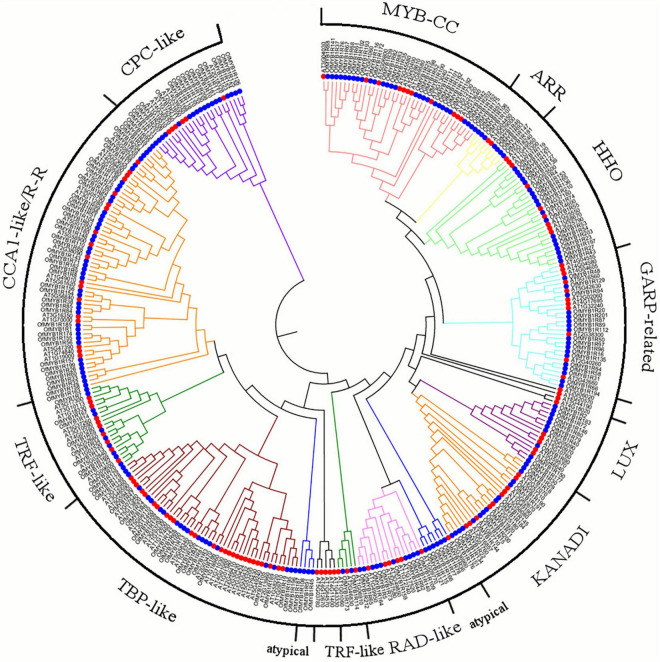
Phylogenetic relationship between the 212 *O. fragrans* (blue circles) and 132 *Arabidopsis* (red circles) *MYB-related* proteins, illustrated by a maximum likelihood (ML) tree. Alignment was performed with the ClustalW program, and these proteins were imported to MEGA 7.0 to construct the phylogenetic tree with 1,000 bootstrap iterations. MYB1Rs represents OfMYB-related proteins.

Additionally, 132 AtMYB-related proteins were analogously identified from the *A. thaliana* genome (Dubos et al., 2010; Liu et al., 2020). The phylogenetic and evolutionary relationships between the 344 AtMYB- and OfMYB-related proteins were analyzed (Figure 1). In accordance with the classification of the MYB-related proteins of Arabidopsis, 212 OfMYB-related proteins were divided into three subgroups: CPC-like, GARP-like (MYB_CC, ARR, HHO, LUX, KANADI, and GRAP-related, considered an unknown subfamily), and a heterogeneous subgroup (consisting of CCA1-like/R-R, TRF-like, TBP-like, RAD-like, and atypical MYB-related proteins).

#### Structure and Motif Analysis of *OfMYB-Related* Genes

**FIGURE 2:**
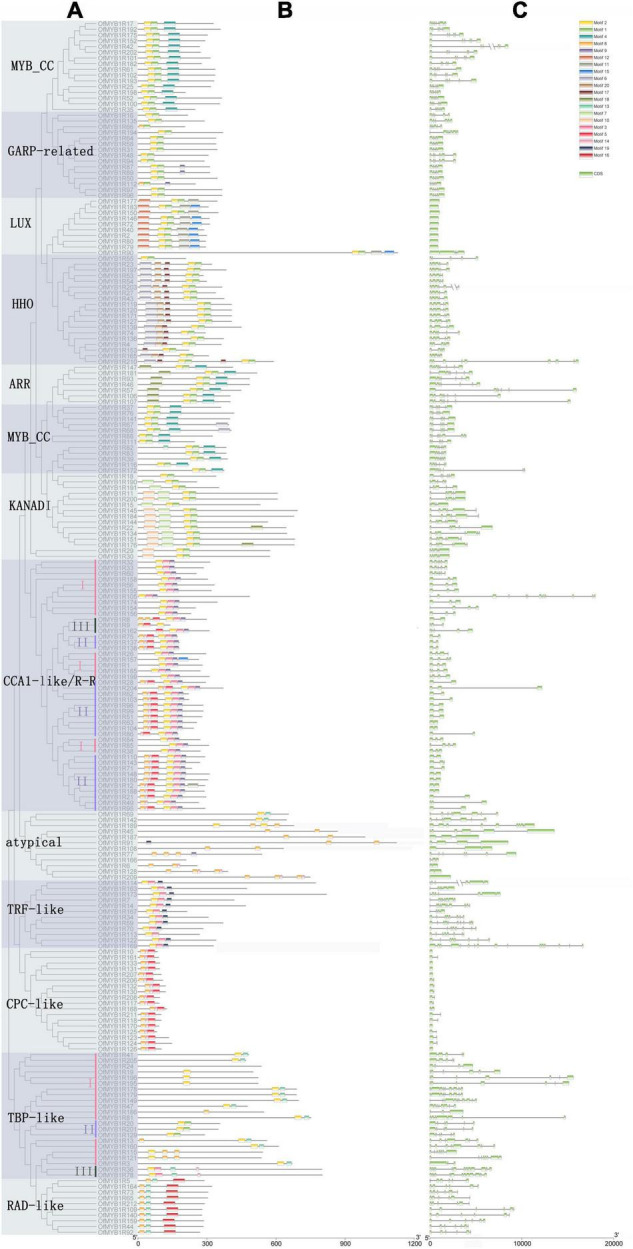
Phylogenetic relationships of 212 OfMYB-related proteins, visualized by a ML tree **(A)**. Conserved motifs of OfMYB-related proteins **(B)**. Exon/intron structures of *OfMYB-related* genes. Exon(s) and intron(s) are shown with green boxes and gray lines, respectively **(C)**. The 20 conserved motifs in the OfMYB-related TFs were predicted by the MEME Suite web server. I–III indicate clades I–III.

The three subgroups, comprising 212 OfMYB-related proteins, were further classified into 12 categories according to highly conserved motifs. The GARP-like subgroup was divided into six clades, whereas the CCA1-like/R-R subgroup consisted of five clades (Figure 2). Moreover, 22 OfMYB-related proteins in the TBP-like subgroup were split into three major clades whose sequence alignments implied high similarity within each clade, thus indicating that these genes may have similar functions.

The largest subgroup was GARP-like, which contained 94 OfMYB-related proteins, comprising MYB_CC, LUX, HHO, KANADI, ARR, and GARP-related, with diversely conserved motifs and CDS patterns (Figure 2); however, the GLK subfamilies did not have BLAST results. The GARP-like subgroup was categorized by the conserved motif Wx3LHx2Fx6L(G)Gx5PK/Sx5Mx4L and the DNA-binding helix 3 domain, harboring the SH(A/L)QK(F/Y) sequence, which is commonly found in diverse plant genomes (Antje et al., 2011). Apart from the common conserved motifs, several unique motifs were detected in different subfamilies, such as the LHEQLE motif in MYB_CC, which contained 28 members and was the largest clade. The smallest clade belonged to ARR, with seven proteins. The second-largest subgroup, with three W residues, consisted of the CCA1-like/R-R, TBP-like, TRF-like, RAD-like, and an atypical MYB-related clade. Notably, the TBP-like subgroup was classified into three distinct clades based on remarkably conserved motifs, and the most common conserved motif of TBP-like was LKDKW(R/K) (N/T) (Supplementary Figure 1). The smallest subgroup, containing only 44 OfMYB-related proteins, was CCA1-like/R-R, an R3-type MYB TF, which had a highly conserved SHAQK(Y/F) (F/Y) motif in the MYB domain third helix—the distinctive characteristic in this subgroup (Supplementary Figure 1).

### Chromosomal Distribution and Gene Synteny Analysis of OfMYB-Related Gene Families

**FIGURE 3:**
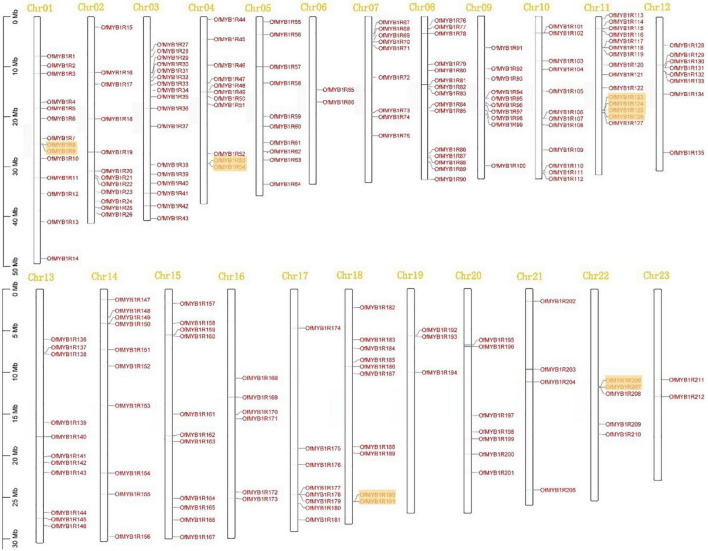
Chromosomal locations of 212 *OfMYB-related* genes, mapped on the basis of the GFF files of *O. fragrans*. The numbers at the top represent each chromosome. Orange boxes represent clusters of tandemly duplicated genes.

The 212 *OfMYB-related* genes were located on all of the 23 uneven sweet osmanthus chromosomes (Figure 3). Remarkably, chromosome (Chr) 03 contained the largest number of *OfMYB-related* genes (17), followed by Chr08 and Chr11 (each with 15 genes), and then Chr01 (14 genes). In contrast, only two genes were mapped to Chr23 and Chr06. The pattern of *OfMYB1Rs* followed a random distribution (sig < 0.05) (Supplementary Figure 2).

Gene duplication plays a crucial role in driving genetic diversity and is common in plant genomes. This research identified seven tandemly duplicated pairs of 12 *OfMYB-related* genes and 68 segmentally duplicated out of the 212 *OfMYB-related* genes. Tandemly duplicated gene pairs were found only in chromosomes 01, 04, 11, 18, and 22. Chr11 had three pairs of tandemly duplicated genes continuously, and segmentally duplicated genes were found on all chromosomes (Figure 3). The tandemly duplicated cluster on Chr11 provides interesting insight into the expansion pattern of sweet osmanthus expansion genes in this large gene family. Moreover, 68 segmental duplication events were distributed across all chromosomes in the synteny blocks (Supplementary Figure 3). Every tandemly duplicated *OfMYB-related* gene pair had a Ka/Ks value less than 1, which evolved in purifying selection; in particular, duplicated gene pairs of *OfMYB1R124* (Chr11) VS. *OfMYB1R125*(Chr11) and *OfMYB1R125* (Chr11) VS. *OfMYB1R126*(Chr11) were significantly different from 1 (Supplementary Table 4). Based on these results, we concluded that some *OfMYB-related* genes originated from gene duplication events, whereas the evolution of the *OfMYB-related* genes was influenced by segmental duplication.

### Expression of OfMYB-Related Genes in Different Organs and Flower Developmental Stages

**FIGURE 4:**
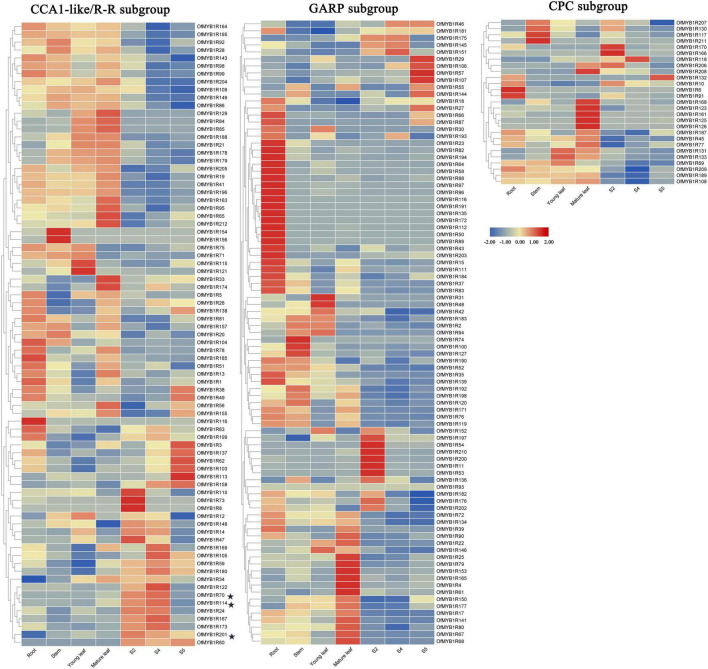
Expression analysis of 194 *OfMYB-related* genes in seven organs. The RPKM values were log_2_ transformed. S2 represents the bud-eye stage, S4 the represents full blooming stage, and S5 represents the flower fading stage. Genes with no transcriptome data are not shown. The candidate genes in the biosynthesis of floral aroma are marked by asterisks.

The expression patterns of *OfMYB-related* genes in different organs and flower developmental stages were analyzed with RNA-sequencing (Supplementary Table 5). Four sweet osmanthus organs were sequenced and used to acquire the reads per kilobase per million mapped reads (RPKM) values of 194 *OfMYB-related* genes, whereas the values of the remaining 17 genes were not found. Eight *OfMYB-related* genes were observed in a single stage: three genes were observed only in the root or primary-bloom stage, two genes (*OfMYB1R74* and *OfMYB1R156*) were observed only in the stem stage. The remaining 186 *OfMYB-related* genes showed different expression patterns, and 142 were present in all samples. Based on the of the conserved motif analysis results, 194 expressed *OfMYB-related* genes were classified into three clear subgroups. Most members of the CCA1-like/R-R subgroup had high expression in leaves as well as the primary- and full-bloom stage of flowers, whereas a wide range of CPC subgroup genes were expressed mainly in the root, stem, and flower fading (S5) stages. The members of the GARP subgroup showed particularly high expression in the root (Figure 4). Remarkably, in the CCA1-like/R-R subgroup, three *OfMYB-related* genes (*OfMYB1R70*, *OfMYB1R114*, and *OfMYB1R201*) had the highest expression in the flower organ/development stage, thus indicating a preferential expression pattern.

### Quantitative Real-Time PCR Analysis

**FIGURE 5:**
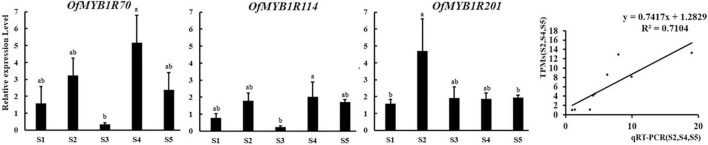
Expression patterns of three candidate *OfMYB-related* genes using the average of three reference genes (*ACTIN*, *RAN*, and *RBP2*) in different stages of sweet osmanthus flowers. Three biological and technological replicate measurements were performed for each stage, and the data are shown as the mean ± SD (*n* = 3) at the top of each bar. The small letters above the error bars indicate significant differences obtained in SPSS 25 with Tukey’s test at *P* < 0.05. S1–S5 represent the bud-pedicel stage, bud-eye stage, primary blooming, full blooming, and flower fading stage, respectively, on the abscissa.

According to the expression patterns and RPKM values of *OfMYB-related* genes (Figure 4), three *OfMYB-related* genes (*OfMYB1R70/114/201*) of the CCA1-like/R-R subgroup were further selected for validation experiments in five flowering stages. The results were reflected those of the RNA-seq experiment, thus verifying the accuracy of the RNA-seq data. The expression of *OfMYB1R70* was similar to that of *OfMYB1R114*, which was generally up-regulated during flowering (S1 and S3). Meanwhile, S3 had the lowest expression, and other processes were down-regulated. Interestingly, the expression of *OfMYB1R70* in S3–S5 was similar to that of *OfMYB1R114*, but the expression of *OfMYB1R70/114* was considerably elevated in stages S3–S4, whereas the expression of *OfMYB1R*201 increased slightly in the same stages (Figure 5). For reliable normalization, we selected three reference genes, *ACTIN*, *RAN*, and *RBP2*, for the qRT-PCR analysis. The expression levels of *OfMYB1Rs* were obtained from the average of three reference genes (Figure 5), and the independent expression results of *ACTIN*, *RAN*, and *RBP2* are shown in Supplementary Figure 4.

### Subcellular Location and Transcriptional Activation Activity of Candidate OfMYB-Related Genes

**FIGURE 6:**
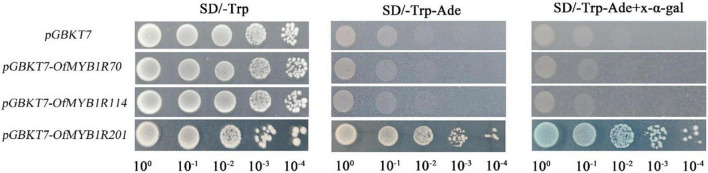
Transcriptional activation analysis of candidate *OfMYB1Rs*. Growth of transformants containing *pGBKT7* and *pGBKT7-OfMYB1R70/114/201* on SD/-Trp medium with serial dilutions. Only *pGBKT7-OfMYB1R201* survived and turned blue on SD/-Trp-Ade medium in the presence of X-A-gal.

To thoroughly examine the potential function of *OfMYB-related* genes in transcriptional regulation, we transformed tobacco leaves to assess the subcellular localization. The GFP fluorescence of the p35S:*OfMYB1R70*:GFP fusion protein was intense in the nucleus, as confirmed by DAPI staining (Supplementary Figure 5). The fluorescence of p35S:*OfMYB1R114*:GFP, like that of the control GFP protein lacking *OfMYB-related* genes, was dispersed in the cytoplasm and nucleus (Supplementary Figure 5). In contrast, the fluorescence of the p35S:*OfMYB1R70/201*:GFP fusion protein was detected in the nucleus and was dispersed in the plasma membrane. To further identify the transcriptional activation functions of the *OfMYB-related* genes, we cloned the CDSs of selected *OfMYB-related* genes into the yeast expression vector pGBKT7. The negative control pGBKT7 and *OfMYB-related* genes showed comparable growth on SD/-Trp medium with serial dilutions (Figure 6). Nevertheless, except for *pGBKT7–*OfMYB1R201, no yeast cells containing *pDEST-GBKT7-OfMYB1R* plasmids survived and turned blue on SD/-Trp-Ade medium with X-α-Gal.

#### Verification and Determination of Volatile Aroma Compounds in Plants With Transient Expression

**FIGURE 7:**
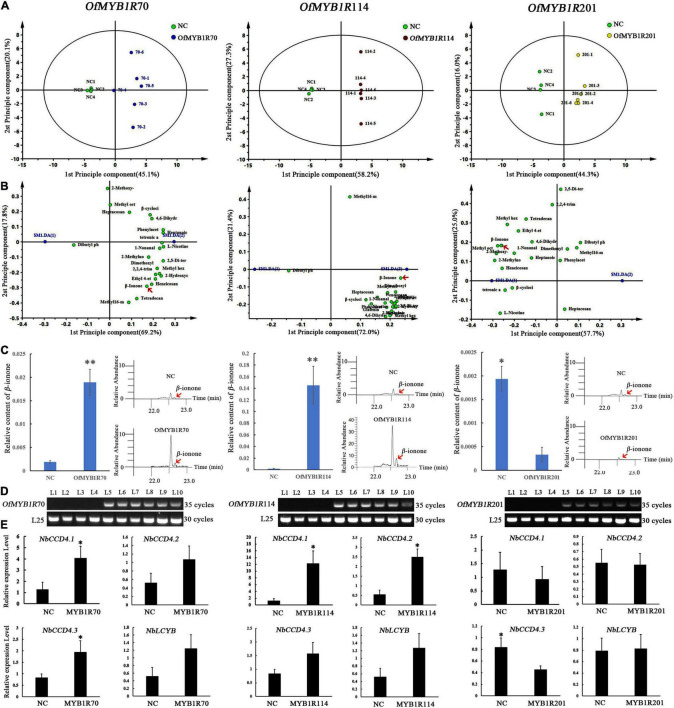
Score scatter plot of the leaf GC-MS profiles of three types plants with transient expression of different *MYB1R* transcription factors in in tobacco, determined with PCA-X analysis in SIMCA13.0 **(A)**. Loading scatter plot analysis of floral VOCs in plants after infiltration of 35S:*OfMYBMYB1R70/114/201*:GFP and control vectors. The red arrow marks the floral aroma in plants with transient expression **(B)**. Floral composition analysis of β-ionone after infiltration with 35S:*OfMYBMYB1R70/114/201*:GFP and control vectors **(C)**. RT-PCR to explore the regulation of gene transcription *OfMYBMYB1R70/114/201* levels. A fragment of the *NbL25* gene was amplified as an internal control. L1–4 represent different plants infiltrated with empty vectors, and L5–10 represent different plants infiltrated with 35S:*OfMYB1R*:GFP vectors **(D)**. qRT-PCR analysis of the NbCCD4 transcript levels in control and transiently transformed tobacco leaves **(E)**. *P < 0.05, ^**^P < 0.01.

To investigate the volatile metabolic components, we obtained the GC-MS total ion current chromatograms of 22 tobacco samples from four types of plants with transient expression. Approximately 23 volatile compounds were identified in the plants with transient expression, including esters, phenolics, ketones, alkanes, and monoterpenes (Supplementary Table 6). PCA-X plots, generated by comparing three types of plants with transient expression and empty vector (NC), showed significant metabolic differences (Figure 7). Notably, according to two principal components, tobacco leaves infiltrated with 35S:*OfMYB1R70/114/201*:GFP differed from those with control vectors in the production of volatile aromatic compounds. *OfMYB1R70/114* and NC clustered separately in the PCA-X. *OfMYB1R201* was slightly scattered in the PCA (Figure 7), thus implying a role in regulating the production of volatile aromatic compounds. Among the three types of plants with transient expression, the plants with transient *OfMYB1R114* expression had a greater ability to regulate aromatics synthesis than the other two types. The comprehensive regulation pattern of *OfMYB1R114* was greater than *OfMYB1R70*, in view of the number of differentially present substances satisfying VIP > 1 and *P* < 0.05.

Interestingly, further analysis revealed that *OfMYB1R114* regulates the synthesis of six esters, two ketones, one monoterpene, one alkane, and one aldehyde, which showed significant discrepancies in the GC-MS analysis of the aroma content with VIP > 1 and SPSS with *P* < 0.05. The substances that slightly changed included floral VOCs such as β-ionone, nonanal, and benzeneacetaldehyde (Supplementary Table 6). Notably, *OfMYB1R114* mainly altered the production of nonanal, which is common in rose and sweet orange, and caused a clear difference in β-ionone. Interestingly, in agreement with the results of correlation analysis, *OfMYB1R114* positively regulated the production of β-cyclocitral. *OfMYB1R70* is predominantly involved in the synthesizing of four esters and two ketones, and the most striking change in floral fragrance compounds was observed for β-ionone, a significant food flavor. In contrast, O*fMYB1R201* showed significantly negative regulation of four esters, one ketone, one aldehyde, and one naphthenic compound, and the production of floral VOCs (nonanal and β-ionone) was comparatively low. Simultaneously, the results of RT-PCR demonstrated that *OfMYB1R70/114/201* were transiently expressed in the plants. *OfMYB1R70/114/201* was detectable in 35S:OfMYB1R:GFP plants with transient expression, whereas *OfMYB1R70/114/201* expression was undetectable in transformed empty vector plants. According to the qRT-PCR results, *NbCCD4.1* was significantly up-regulated in plants with transient expression of *OfMYB1R70* and *OfMYB1R114*, and *NbCCD4.2* was markedly elevated in the plants with transient expression of *OfMYB1R114*. Interestingly, *NbCCD4.3* was significantly elevated in the plants with transient expression of *OfMYB1R70* and dramatically diminished in the plants with transient expression of *OfMYB1R201* (Figure 7). These results indicated that the transient overexpression of *OfMYB1R70* activated the expression of *NbCCD4.1* and *NbCCD4.2*; *OfMYB1R114* might have activated *NbCCD4.1* and *NbCCD4.3*; and *OfMYB1R201* repressed the transcription of *NbCCD4.3* in tobacco plants, thus leading to the changes in β-ionone content in the plants with transient expression of the three *OfMYB1Rs*.

## Discussion

To date, MYB TFs, mainly comprising MYB-related and R2R3 MYB, modulate the transcription levels of numerous essential genes in secondary metabolism biosynthetic pathways, thereby regulating the production of diverse floral VOCs (Jian et al., 2019). Several MYB members have been identified as a conserved module regulating terpene synthesis. *MsMYB*, a novel negative regulator in *Mentha spicata* associated with the promoter region of geranyl diphosphate synthase (GPPS), decreases the production of monoterpenes by inhibiting the activity of GPPS (Reddy et al., 2017). Recently, the function of R2R3MYBs in floral aroma formation in *Hedychium coronarium* has been extensively reported (Abbas et al., 2021). However, further studies of MYB-related proteins, particularly the close relationship between the function of *MYB-related* TFs and floral aroma synthesis, remain unclear.

The number of *OfMYB-related* genes was more significant than that observed in other plants reported over the recent years (Table 1; Jin et al., 2017), such as 109 genes in peach (Zhang et al., 2018). In our study, we identified 212 *MYB-related* genes in *O. fragrans*, a value close to the number of *R2R3-MYB* genes in *O. fragrans* (243 genes) (Li et al., 2020a), thus representing the largest subfamily of *MYB* genes, which play critical roles in various processes of growth, development, and responses to abiotic or biotic stress. While high, the number of *R2R3-MYB* subfamily members did not exceed the number of *1R-MYB* subfamily members. The amount of *1R-MYBs* in *Ganoderma* was twice the number of *R2R3-MYBs*. Moreover, previous studies have performed genome-wide analyses of MYB-related proteins from 16 species of flowering plants, moss, Selaginella, and algae (Du et al., 2013). In contrast to the detailed studies on the *R2R3-MYB* subfamily, little attention has been paid to the *MYB-related* (*1R-MYB*) subfamily, which has been reported only in a few common crops in recent years: potato, sunflower (*Helianthus annuus*), radish (*Raphanus sativus*), and oil palm (*Elaeis guineensis*) (Li et al., 2020b; Liu et al., 2020; Zhou et al., 2020; Muleke et al., 2021).

As an important part of the *MYB* family, *MYB*-related genes were initially classified into five subgroups: the CCA1-like, CPC-like, TBP-like, I-box-binding-like, R-R-type (Zhang et al., 2018). However, after detailed studies on the GARP family, *MYB*-related genes were divided into three subgroups: CCA1-like/R-R, CPC-like, and GARP-like (Liu et al., 2020). After extensive further studies, a merger occurred in the Circadian Clock Associated 1-like and R-R-type, equivalent to the CCA1-like/R-R subgroups in this study; the results are consistent with those of the most recent studies (Liu et al., 2020). In previous work, CCA1-like proteins have been found to be closely associated with R-R type MYB repeats, thus implying that R-R-type gene loss events may directly lead to CCA1-like proteins. In this classification, 44 members of the CCA1-like/R-R group contained the conserved motif SHAQK (motif 2 in Supplementary Figure 1) in the MYB repeat, in agreement with previous studies in Arabidopsis (Ji et al., 2019). The CAPR-like subgroups initially comprised maize G2-like and Arabidopsis ARR-B, which were newly derived in angiosperms. In agreement with previous research, the GARP-like subgroups were classified into six clades in this identification (Liu et al., 2020).

As a typical MYB member, the CPC-like subgroup has been reported to be involved in the basic function of regulating root-hair development and the plant trichome. It has been confirmed to regulate the synthesis of secondary metabolites in recent years. For instance, *AtCPC*, characterized as a repressor of anthocyanin production, has provided novel insight into the transcriptional regulation of flavonoid biosynthesis. *SlTRY* and *SlGL3*, *MYB-related* homologs of tomato, also influence synthesis of anthocyanin (Jian et al., 2019). In contrast, *AtMYBL2*, an R3-MYB protein, has been found to restrain flavonoid synthesis. Interestingly, *MYBL2* inhibits flavonoid biosynthesis and trichome development (Matsui et al., 2008). *LlMYB3*, a *MYB* related homolog from tiger lily, positively regulates the stress tolerance of plants and is involved in the anthocyanin biosynthesis pathway, thereby regulating stress tolerance (Yong et al., 2019).

In this expression analysis, *OfMYB1R70*, *OfMYB1R114*, and *OfMYB1R201*, which were preferentially expressed in floral organs, had higher RPKM values than other *OfMYB1Rs*, particularly *OfMYB1R70* and *OfMYB1R114*, which had the top two RPKM values. Moreover, subcellular localization prediction indicated that they were mainly located in the nucleus, thus suggesting that these candidate genes may play important roles in regulating floral aroma synthesis during the bloom-stage of sweet osmanthus; therefore, they were selected for follow-up experiments. However, no prior studies have focused on the functions of MYB-related TFs in floral aroma biosynthesis. The relationship with the synthesis of floral VOCs has been reported only for 2RMYB (Reddy et al., 2017), although *2RMYB* and *MYB-related* TFs jointly compose the main family in plants and are involved in many biological processes, particularly secondary metabolism, and responses to environmental factors, in divergent ways (Jian et al., 2019; Zhao et al., 2019).

Transcription factors have been reported to regulate the synthesis of floral VOCs by directly binding the promoter regions of target enzyme genes and controlling their expression levels. In *H. coronarium*, *HcMYB* genes directly bind the promoters of key structural volatile synthesis genes and regulate the biosynthesis of terpenoids and benzenoids (Abbas et al., 2021). In *O. fragrans*, *OfERF61* up-regulates the expression of *OfCCD4* and influences the synthesis of β-ionone (Han et al., 2019). In petunia, *PhMYB4* controls the production of floral volatile benzenoid/phenylpropanoid by suppressing the downstream gene transcription (Thomas et al., 2011). *PhERF6* binds the promoters of EOBI protein, which influences floral scent, thus negatively regulating the production of volatile compounds (Liu et al., 2017). In spearmint, *MsMYB* represses the expression of the *GPPS* gene, thus modifying secondary terpene metabolism (Reddy et al., 2017). In this study, we found that *OfMYB1R201* is nuclearly located with self-activation activity its transient overexpression decreases the synthesis of β-ionone (Figure 7) and other VOCs in tobacco leaves (Supplementary Figure 6). The expression of *NbCCD4.3* was significantly diminished in transformed plants with transient expression of the *OfMYB1R201* vector (Figure 7). These results indicated that *OfMYB1R201* could participate in negatively regulating VOC synthesis.

Remarkably, *OfMYB1R70* and *OfMYB1R114* were found to have nuclear localization (Supplementary Figure 5), and their expression during flower development was highly correlated (Figure 4). The transient transformation results showed that *OfMYB1R70* and *OfMYB1R114* were both involved in the regulation of aroma component synthesis, as confirmed by the markedly greater production of β-ionone and other emitted metabolites than that in the empty vector plants (Supplementary Figure 6). The expression of *NbCCD4.1* and *NbCCD4.3* was elevated in plants with transient expression of the *OfMYB1R114* vector, and the expression of *NbCCD4.1* and *NbCCD4.2* was markedly elevated in plants with transient expression of the *OfMYB1R70* vector (Figure 7). However, we found that *OfMYB1R70* and *OfMYB1R114* had no transcriptional activity, thus indicating that they do not directly activate the transcription of their downstream genes. Previous studies have reported that TFs interact with other proteins in regulating the synthesis of secondary metabolites by the MBW complex (Koes et al., 2005). For example, *Fh*MYBx, containing a bHLH-binding motif in the R3 domains, suppresses the formation of the MYB-bHLH-WD40 (MBW) complex via an association with bHLH proteins, thus down-regulating anthocyanin metabolism (Li et al., 2020c). *SmelMYBL1*, an R3 MYB repressor in eggplant, acts as an inhibitor of the MBW complex by competing with MYB activators (*SmelANT1* and *SmelAN2*) for the bHLH binding site involved in anthocyanin biosynthesis (Andrea et al., 2020). Hence, we concluded that *OfMYB1R70*/*114/201* regulates aroma component formation by interacting with other TFs or acting as activators in competing pathways that alter the availability of precursors. The molecular mechanism of *OfMYB1R70/114/201* in regulating the aroma metabolite synthesis will be further explored in following studies.

## Conclusion

In this study, to provide novel insight into the regulation of floral aroma by all *MYB-related* genes in sweet osmanthus, we comprehensively identified 212 putative *MYB-related* members by genome-wide analysis. We categorized them into three subgroups, including 12 categories related to 132 *MYB-related* members of *A. thaliana* in the phylogenetic tree. Additionally, the information on conserved motifs in genes was visualized in figures, and the expression patterns indicated that three candidate *OfMYB1Rs* might be associated with the development and growth of flowers. Notably, members of the CC1-like/R-R subgroup in sweet osmanthus, *OfMYB1R70/114/201*, localized primarily in the nucleus. Remarkably, transient expression studies demonstrated that all those genes were associated with the regulation of floral VOC synthesis, and *OfMYB1R114* had an apparent ability to positively regulate the synthesis of floral VOCs, such as β-ionone and nonanal, whereas *OfMYB1R201*, as a TF, negatively regulated floral VOCs. Together, our findings provide insight into *OfMYB-related* genes, explaining their roles in plants as regulators of the production of volatile aroma compounds. Our future efforts will focus on modulating the functions of *OfMYB1R70/114/201* in regulating the production of floral aroma and investigating the roles of upstream regulatory factors.

## Data Availability Statement

The original contributions presented in the study are included in the article/Supplementary Material, further inquiries can be directed to the corresponding authors.

## Author Contributions

YY and XnY conceived the original idea. XnY and XW performed the experimental work. XnY and WD contributed to the data analysis. XnY wrote the manuscript. All authors contributed to the article and approved the submitted version.

## Conflict of Interest

The authors declare that the research was conducted in the absence of any commercial or financial relationships that could be construed as a potential conflict of interest.

## Publisher’s Note

All claims expressed in this article are solely those of the authors and do not necessarily represent those of their affiliated organizations, or those of the publisher, the editors and the reviewers. Any product that may be evaluated in this article, or claim that may be made by its manufacturer, is not guaranteed or endorsed by the publisher.

## Footnotes

1
http://www.arabidopsis.org/

2
http://meme-suite.org/tools/meme

3
http://mg2c.iask.in/mg2c_v2.0/

4
https://sourceforge.net/projects/mcscanx/

5
https://webbook.nist.gov/

## Supplementary Material

Supplementary Figure 1 Sequence logos of the MYB-related domains of subgroups.

Supplementary Figure 2 The classification and frequency of each subgroup on each chromosome.

Supplementary Figure 3 Tandemly and segmentally duplicated genes visualized in the *O. fragrans* genome.

Supplementary Figure 4 The expression patterns of three candidate *OfMYB1Rs*, on the basis of three reference genes (*ACTIN*, *RAN*, and *RBP2*).

Supplementary Figure 5 Subcellular localization of candidate OfMYB-related proteins.

Supplementary Figure 6 Floral composition analysis after infiltration with 35S:*OfMYBMYB1R70/114/201*:GFP and control vectors (VIP > 1 and *P* < 0.05).

Supplementary Figure 7 OPLS-DA plots generated from comparisons among three types of plants with transient expression and negative control vector plants with transient expression (NC).

Supplementary Figure 8 The process of transient expression in *N. benthamiana*.
