# Supplementary figures and images for: Insights Into the MYB-Related Transcription Factors Involved in Regulating Floral Aroma Synthesis in Sweet Osmanthus
Source: Front Plant Sci. 2022 Mar 9;13:765213. doi: 10.3389/fpls.2022.765213 (PMC8959829; doi:10.3389/fpls.2022.765213)

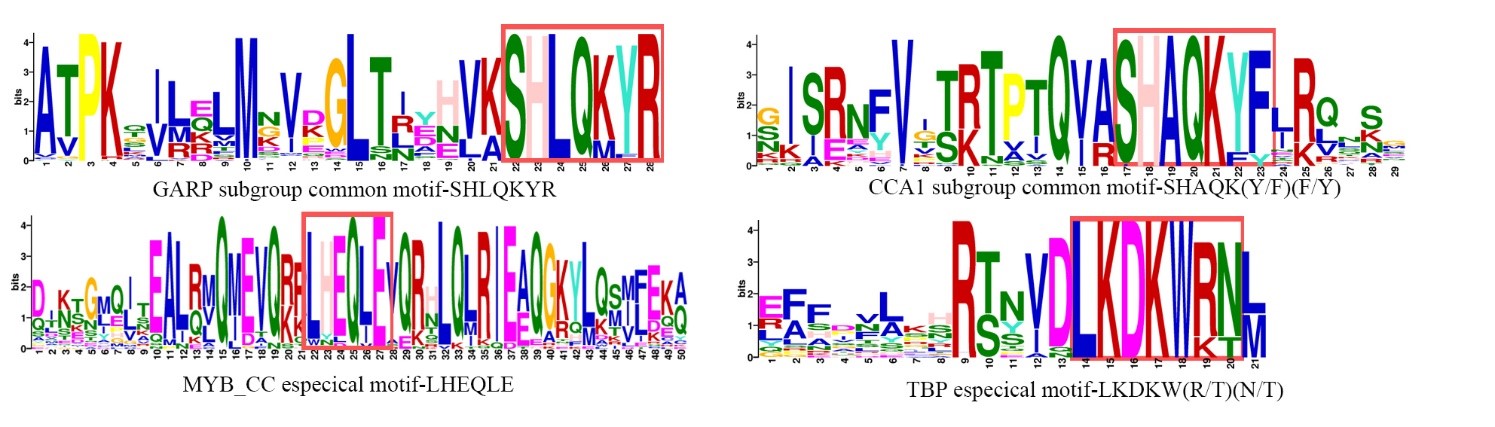

Supplement: Supplementary Figure 1 — Sequence logos of the MYB-related domains of subgroups. [file Image_1.JPEG]

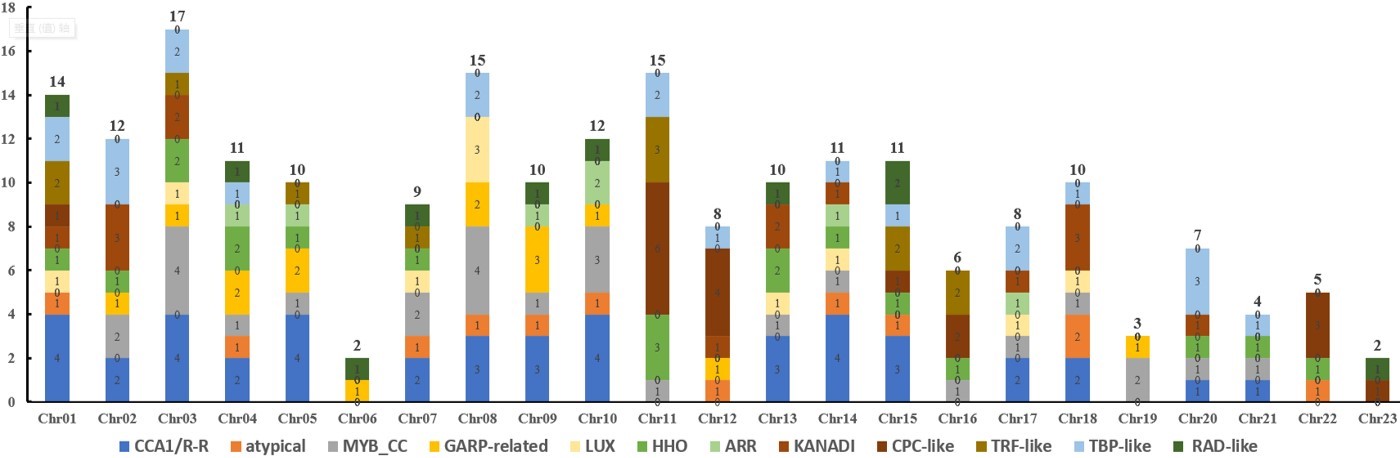

Supplement: Supplementary Figure 2 — The classification and frequency of each subgroup on each chromosome. [file Image_2.JPEG]

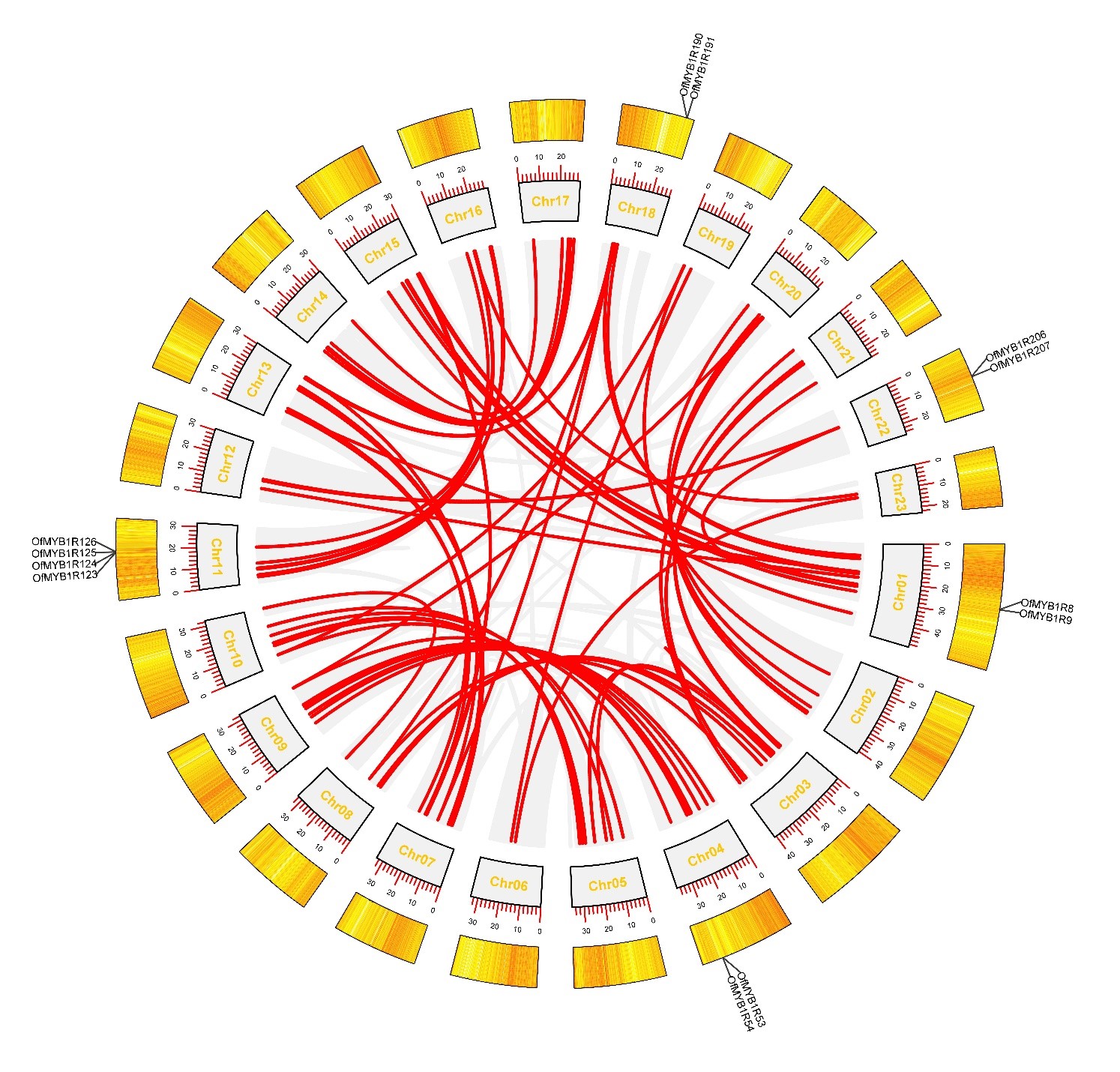

Supplement: Supplementary Figure 3 — Tandemly and segmentally duplicated genes visualized in the O. fragrans genome. [file Image_3.JPEG]

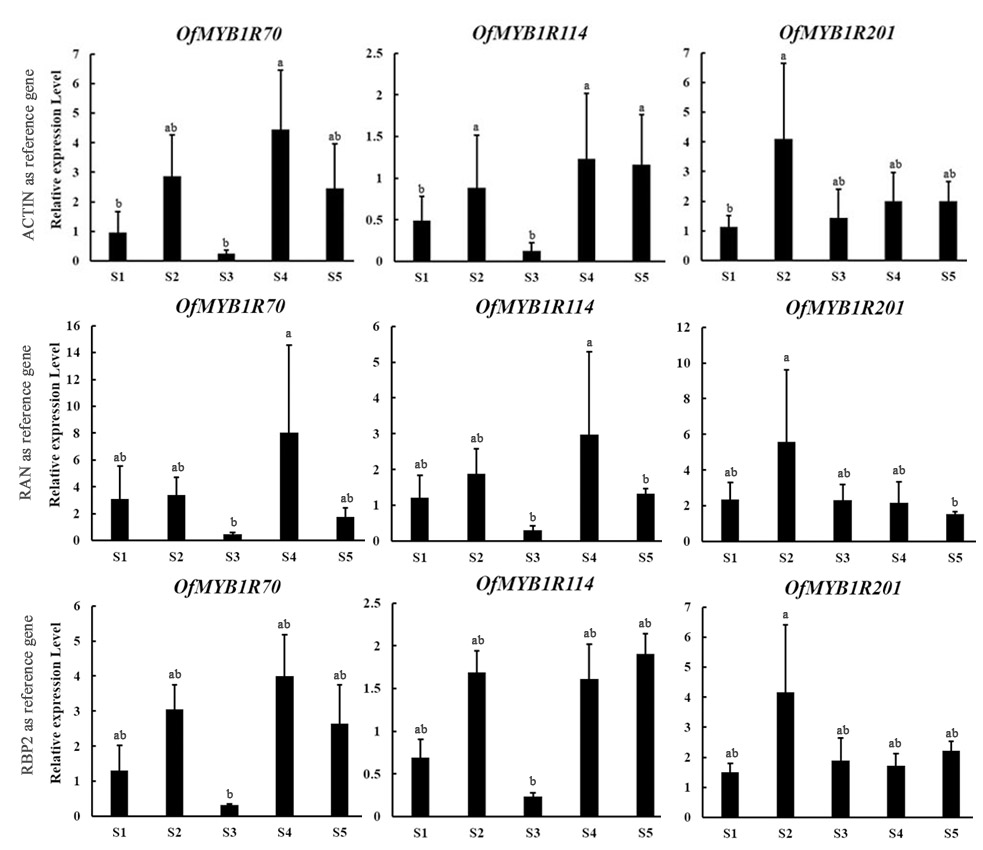

Supplement: Supplementary Figure 4 — The expression patterns of three candidate OfMYB1Rs, on the basis of three reference genes (ACTIN, RAN, and RBP2). [file Image_4.JPEG]

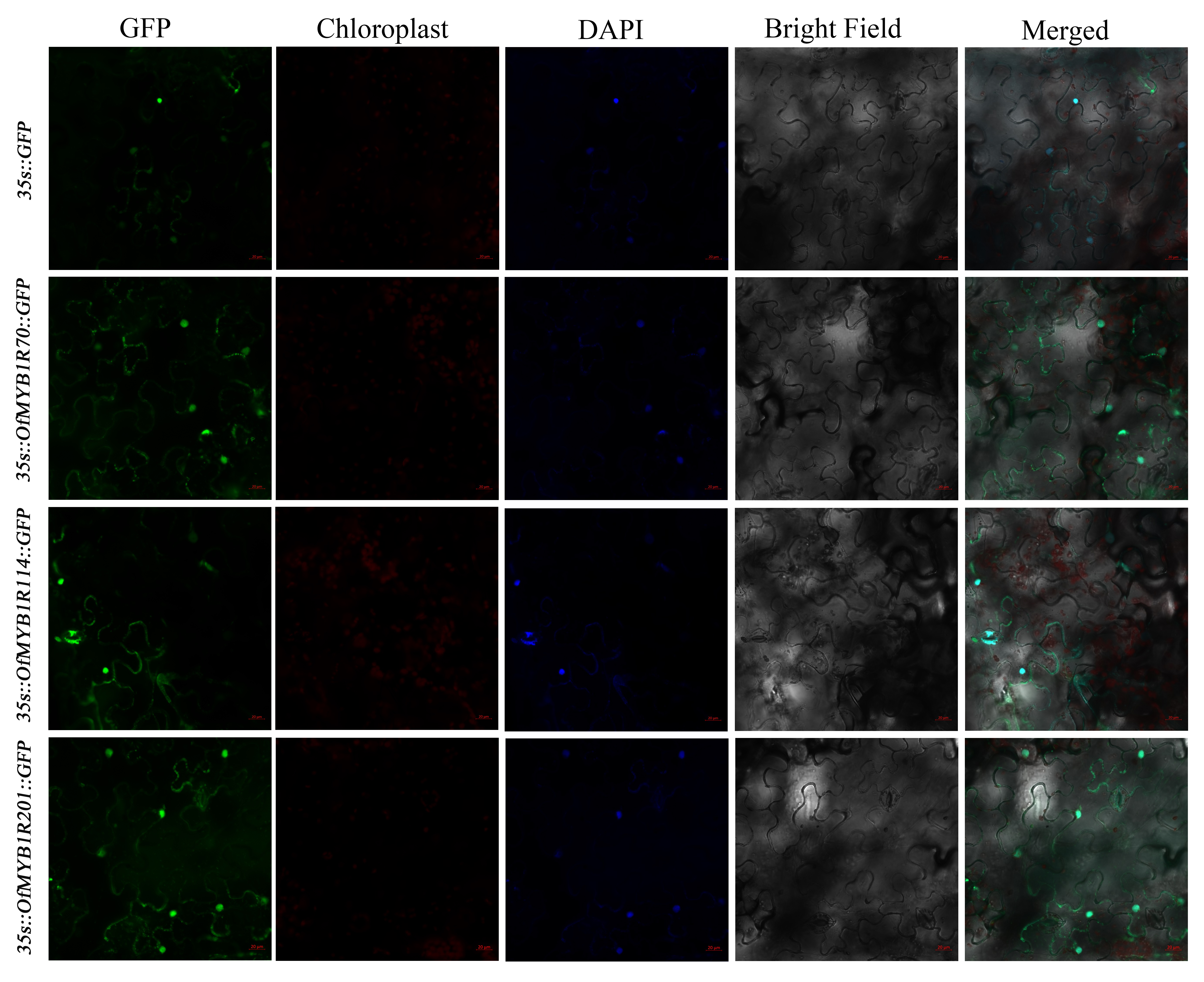

Supplement: Supplementary Figure 5 — Subcellular localization of candidate OfMYB-related proteins. [file Image_5.JPEG]

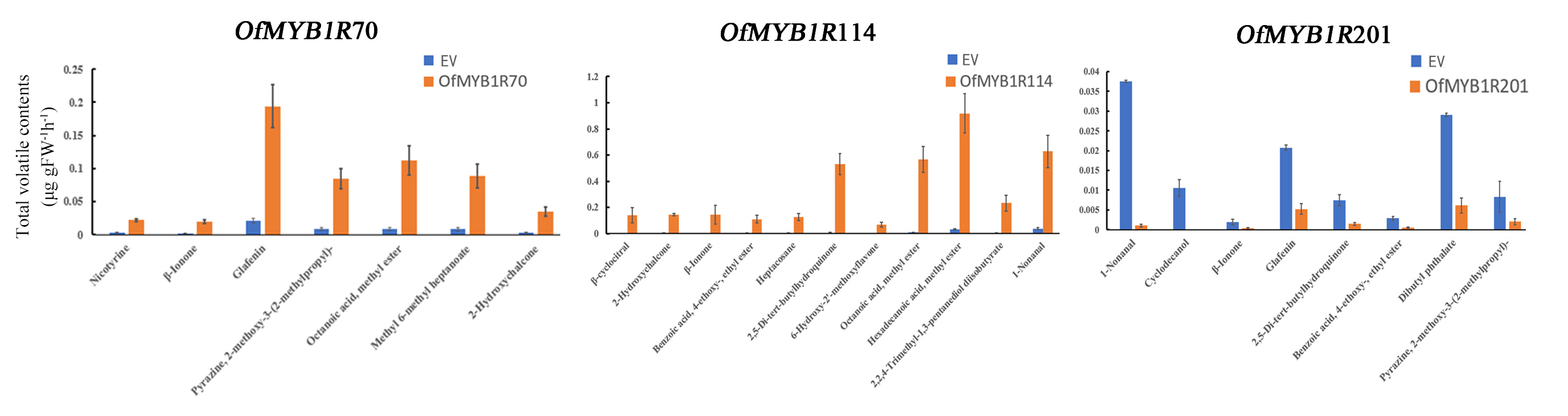

Supplement: Supplementary Figure 6 — Floral composition analysis after infiltration with 35S:OfMYBMYB1R70/114/201:GFP and control vectors (VIP > 1 and P < 0.05). [file Image_6.JPEG]

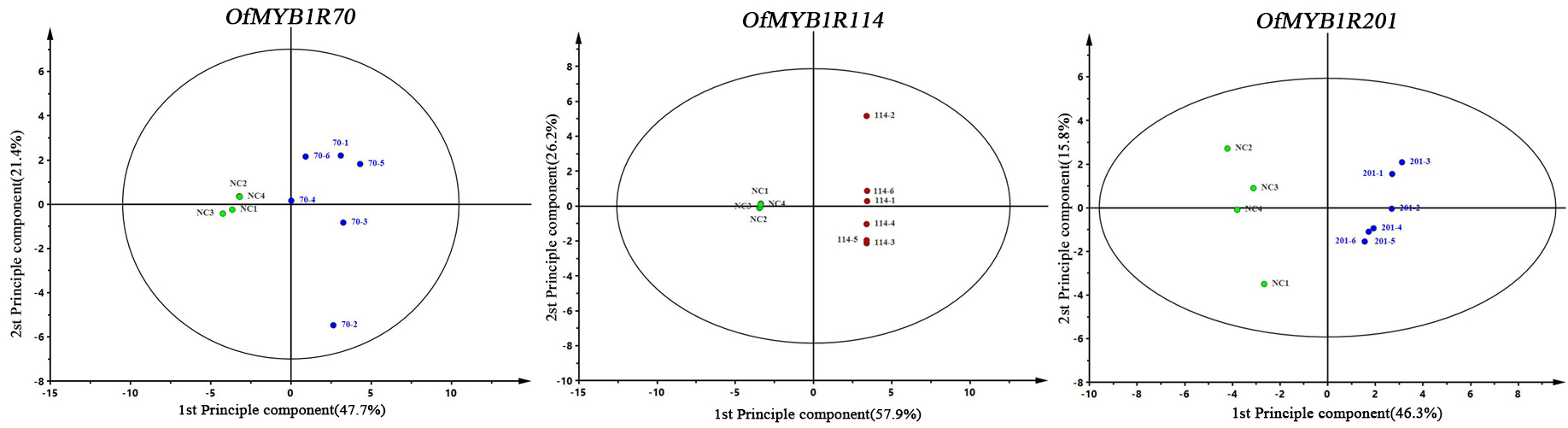

Supplement: Supplementary Figure 7 — OPLS-DA plots generated from comparisons among three types of plants with transient expression and negative control vector plants with transient expression (NC). [file Image_7.JPEG]

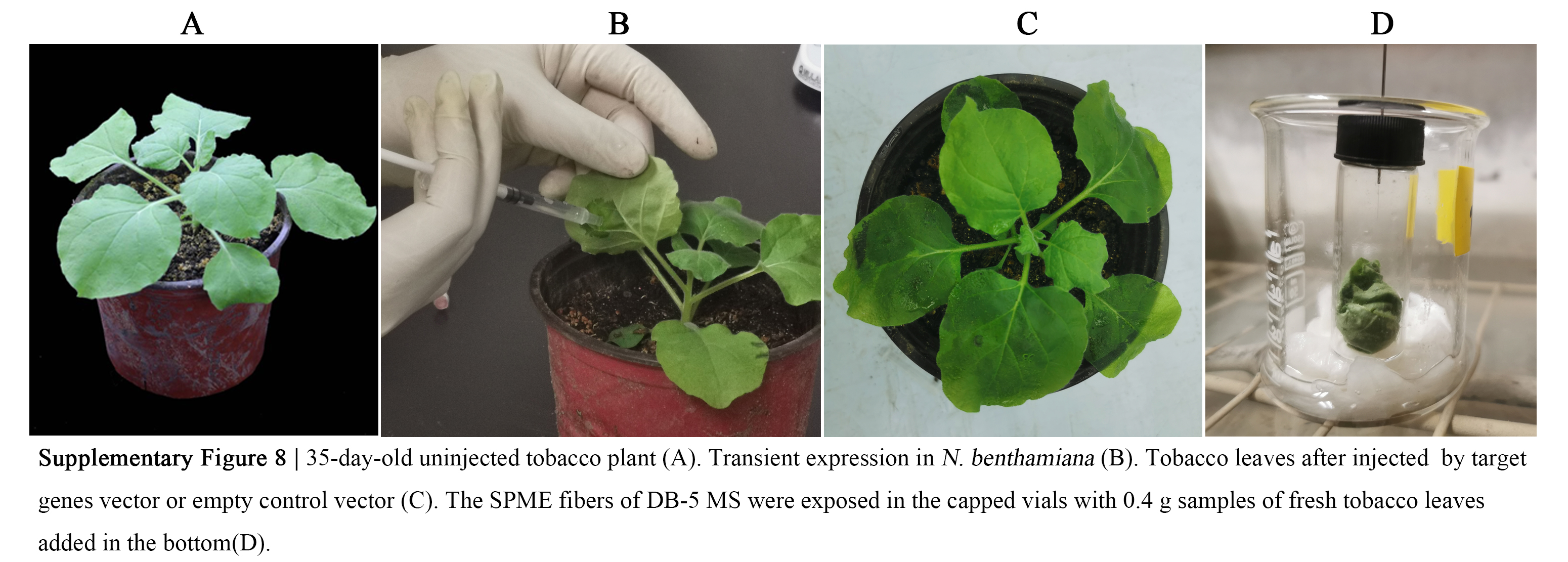

Supplement: Supplementary Figure 8 — The process of transient expression in N. benthamiana. [file Image_8.JPEG]
