# Supplementary material for: Insights Into the MYB-Related Transcription Factors Involved in Regulating Floral Aroma Synthesis in Sweet Osmanthus
Source: Front Plant Sci. 2022 Mar 9;13:765213. doi: 10.3389/fpls.2022.765213 (PMC8959829; doi:10.3389/fpls.2022.765213)
Supplement: Supplementary file 10 [file Table_1.DOCX]

The primers used for quantitative real-time PCR (qRT-PCR) and semi-quantitative RT-PCR (RT-PCR)

| Gene | Primer name | Primer sequences |
| --- | --- | --- |
| *Of*ACTIN | F | CCCAAGGCAAACAGAGAAAAAAT |
|  | R | ACCCCATCACCAGAATCAAGAA |
| *OfMYB1R70* | F | TAAAGGCCATGTGCAGAAGC |
|  | R | AAAGTCTGGGCTGGACAAGTT |
| *OfMYB1R114* | F | TCCGACTTCATCTACTGCTTCTG |
|  | R | TCTTGATTGGGGATTGGGTT |
| *OfMYB1R201* | F | ACCCTCCAGCCTTCCCATT |
|  | R | AACCTCGAAAGAAACCTTGACC |
| *NbCCD4.1* | F | TACCACCAAACAAACAGTAGAGC |
|  | R | TCAATGAAAGCGTTCACGAAA |
| *NbCCD4.2* | F | ACAAGAAAAGCCAACCCCATC |
|  | R | TGGAAATGATGGCCCTACTGT |
| *NbCCD4.3* | F | ACGGTTTCCACGGGCTTTT |
|  | R | GATGACACCCATGCCCTCTT |
| *NbLCYB* | F | TGGCGGAAGTGGAGGAAC |
|  | R | TACCCAAGTGACTTAAACGAGCC |
| *NbL25* | F | GCTAAGGTTGCCAAGGCTGTC |
|  | R | TAAGGTATTGACTTTCTTTGTCTGA |
