# Supplementary material for: Insights Into the MYB-Related Transcription Factors Involved in Regulating Floral Aroma Synthesis in Sweet Osmanthus
Source: Front Plant Sci. 2022 Mar 9;13:765213. doi: 10.3389/fpls.2022.765213 (PMC8959829; doi:10.3389/fpls.2022.765213)
Supplement: Supplementary file 11 [file Table_2.DOCX]

Detailed information about the *OfMYB-related* transcription factors

| Gene ID | Rename | Start position | End position | CDS Length | PI | MW(Da) | Location |
| --- | --- | --- | --- | --- | --- | --- | --- |
| evm.model.Contig106.66 | OfMYB1R1 | 7874977 | 7876697 | 1720 | 7.99 | 30240.65 | Chr01 |
| evm.model.Contig167.116 | OfMYB1R2 | 9738532 | 9739431 | 899 | 6.14 | 32357.3 | Chr01 |
| evm.model.Contig60.89 | OfMYB1R3 | 11366636 | 11369392 | 2756 | 6.51 | 75454.61 | Chr01 |
| evm.model.Contig71.216 | OfMYB1R4 | 17189555 | 17191648 | 2093 | 8.92 | 42029.68 | Chr01 |
| evm.model.Contig71.92 | OfMYB1R5 | 18317368 | 18321579 | 4211 | 8.91 | 32056.67 | Chr01 |
| evm.model.Contig66.106 | OfMYB1R6 | 20356850 | 20357700 | 850 | 5.69 | 29235.69 | Chr01 |
| evm.model.Contig166.72 | OfMYB1R7 | 24302880 | 24305633 | 2753 | 6.06 | 46746.58 | Chr01 |
| evm.model.Contig213.15 | OfMYB1R8 | 25491600 | 25493234 | 1634 | 4.93 | 34342.35 | Chr01 |
| evm.model.Contig213.17 | OfMYB1R9 | 25518957 | 25520443 | 1486 | 4.87 | 16461.43 | Chr01 |
| evm.model.Contig283.19 | OfMYB1R10 | 28367805 | 28368062 | 257 | 9.1 | 9915.14 | Chr01 |
| evm.model.Contig41.4 | OfMYB1R11 | 32247465 | 32251316 | 3851 | 5.96 | 69224.59 | Chr01 |
| evm.model.Contig6.17 | OfMYB1R12 | 35475817 | 35476799 | 982 | 8.35 | 31922 | Chr01 |
| evm.model.Contig50.105 | OfMYB1R13 | 41060274 | 41065525 | 5251 | 6.08 | 62609.11 | Chr01 |
| evm.model.Contig209.37 | OfMYB1R14 | 48381224 | 48385574 | 4350 | 6.1 | 52400.05 | Chr01 |
| evm.model.Contig28.92 | OfMYB1R15 | 2101914 | 2103892 | 1978 | 5.89 | 59953.72 | Chr02 |
| evm.model.Contig70.102 | OfMYB1R16 | 11142374 | 11144505 | 2131 | 10.1 | 25245.68 | Chr02 |
| evm.model.Contig620.8 | OfMYB1R17 | 13508232 | 13509974 | 1742 | 9.12 | 36927.18 | Chr02 |
| evm.model.Contig52.46 | OfMYB1R18 | 20440809 | 20443480 | 2671 | 5.74 | 37390.84 | Chr02 |
| evm.model.Contig282.18 | OfMYB1R19 | 27084153 | 27091781 | 7628 | 8.2 | 54108.43 | Chr02 |
| evm.model.Contig45.169 | OfMYB1R20 | 30885999 | 30890837 | 4838 | 7.81 | 40583.2 | Chr02 |
| evm.model.Contig196.125 | OfMYB1R21 | 31854005 | 31858334 | 4329 | 7.18 | 31673.37 | Chr02 |
| evm.model.Contig196.85 | OfMYB1R22 | 32225169 | 32231964 | 6795 | 5.96 | 70625.36 | Chr02 |
| evm.model.Contig206.46 | OfMYB1R23 | 35057587 | 35059574 | 1987 | 8.5 | 35192.29 | Chr02 |
| evm.model.Contig129.54 | OfMYB1R24 | 36949983 | 36954654 | 4671 | 4.77 | 60676.28 | Chr02 |
| evm.model.Contig210.43 | OfMYB1R25 | 38204412 | 38205886 | 1474 | 6.77 | 36146.77 | Chr02 |
| evm.model.Contig269.47 | OfMYB1R26 | 39280072 | 39282049 | 1977 | 9.8 | 33070.02 | Chr02 |
| evm.model.Contig186.89 | OfMYB1R27 | 6739521 | 6741355 | 1834 | 9.14 | 37899.33 | Chr03 |
| evm.model.Contig37.265 | OfMYB1R28 | 7959484 | 7962317 | 2833 | 6.83 | 31670.79 | Chr03 |
| evm.model.Contig300.97 | OfMYB1R29 | 11160661 | 11162753 | 2092 | 5.46 | 65509.06 | Chr03 |
| evm.model.Contig35.10 | OfMYB1R30 | 11296308 | 11298417 | 2109 | 5.52 | 65556.16 | Chr03 |
| evm.model.Contig35.91 | OfMYB1R31 | 12112889 | 12114325 | 1436 | 8.33 | 38977.2 | Chr03 |
| evm.model.Contig35.183 | OfMYB1R32 | 12855637 | 12857531 | 1894 | 9.56 | 34250.69 | Chr03 |
| evm.model.Contig35.291 | OfMYB1R33 | 13656767 | 13658625 | 1858 | 9.59 | 31435.51 | Chr03 |
| evm.model.Contig64.205 | OfMYB1R34 | 14784724 | 14788441 | 3717 | 9.22 | 33828.36 | Chr03 |
| evm.model.Contig64.47 | OfMYB1R35 | 15976326 | 15977923 | 1597 | 9.04 | 27820.62 | Chr03 |
| evm.model.Contig337.24 | OfMYB1R36 | 18331352 | 18338060 | 6708 | 7.22 | 88409.23 | Chr03 |
| evm.model.Contig222.52 | OfMYB1R37 | 21888376 | 21890784 | 2408 | 6.92 | 40876.7 | Chr03 |
| evm.model.Contig48.24 | OfMYB1R38 | 29654240 | 29655529 | 1289 | 9.39 | 29457.13 | Chr03 |
| evm.model.Contig131.46 | OfMYB1R39 | 31525067 | 31526744 | 1677 | 8.15 | 43429.88 | Chr03 |
| evm.model.Contig111.33 | OfMYB1R40 | 33319296 | 33320159 | 863 | 6.62 | 31255.14 | Chr03 |
| evm.model.Contig87.37 | OfMYB1R41 | 35322274 | 35325958 | 3684 | 6.57 | 54704.77 | Chr03 |
| evm.model.Contig543.11 | OfMYB1R42 | 37859544 | 37876969 | 17425 | 7.65 | 29269.46 | Chr03 |
| evm.model.Contig624.9 | OfMYB1R43 | 40349130 | 40351041 | 1911 | 8.65 | 41707.35 | Chr03 |
| evm.model.Contig53.279 | OfMYB1R44 | 519456 | 523635 | 4179 | 9.4 | 31882.67 | Chr04 |
| evm.model.Contig33.258 | OfMYB1R45 | 4474039 | 4487587 | 13548 | 5.69 | 199697 | Chr04 |
| evm.model.Contig138.55 | OfMYB1R46 | 9687189 | 9692642 | 5453 | 5.14 | 53393.36 | Chr04 |
| evm.model.Contig328.41 | OfMYB1R47 | 13238378 | 13241176 | 2798 | 9.81 | 52944.04 | Chr04 |
| evm.model.Contig94.84 | OfMYB1R48 | 14676426 | 14679261 | 2835 | 6.76 | 34753.11 | Chr04 |
| evm.model.Contig94.115 | OfMYB1R49 | 15047425 | 15053579 | 6154 | 7.84 | 28205.05 | Chr04 |
| evm.model.Contig437.42 | OfMYB1R50 | 16017217 | 16018710 | 1493 | 6.65 | 39617.8 | Chr04 |
| evm.model.Contig305.36 | OfMYB1R51 | 17464907 | 17466420 | 1513 | 9.33 | 31615.56 | Chr04 |
| evm.model.Contig205.56 | OfMYB1R52 | 27446525 | 27448065 | 1540 | 6.62 | 41160.17 | Chr04 |
| evm.model.Contig69.76 | OfMYB1R53 | 29372233 | 29373630 | 1397 | 8.9 | 32365.44 | Chr04 |
| evm.model.Contig69.77 | OfMYB1R54 | 29391789 | 29393238 | 1449 | 8.66 | 33515.58 | Chr04 |
| evm.model.Contig273.57 | OfMYB1R55 | 993076 | 998300 | 5224 | 9.43 | 24200.22 | Chr05 |
| evm.model.Contig221.24 | OfMYB1R56 | 3542802 | 3545750 | 2948 | 7.69 | 35957.15 | Chr05 |
| evm.model.Contig272.17 | OfMYB1R57 | 9979956 | 9995892 | 15936 | 6.36 | 50227.49 | Chr05 |
| evm.model.Contig434.4 | OfMYB1R58 | 13270587 | 13272001 | 1414 | 8.38 | 38111.19 | Chr05 |
| evm.model.Contig227.70 | OfMYB1R59 | 19935648 | 19940354 | 4706 | 9.47 | 40822.19 | Chr05 |
| evm.model.Contig170.1 | OfMYB1R60 | 21928131 | 21929809 | 1678 | 9.48 | 28106.88 | Chr05 |
| evm.model.Contig78.139 | OfMYB1R61 | 25213087 | 25216491 | 3404 | 6.87 | 36580 | Chr05 |
| evm.model.Contig86.69 | OfMYB1R62 | 26964048 | 26965579 | 1531 | 6.67 | 25214.12 | Chr05 |
| evm.model.Contig235.33 | OfMYB1R63 | 28645055 | 28645896 | 841 | 9.61 | 29179.2 | Chr05 |
| evm.model.Contig296.15 | OfMYB1R64 | 33502897 | 33504310 | 1413 | 8.89 | 38030.12 | Chr05 |
| evm.model.Contig112.70 | OfMYB1R65 | 14586953 | 14591305 | 4352 | 9.47 | 32912.29 | Chr06 |
| evm.model.Contig388.50 | OfMYB1R66 | 17070976 | 17072274 | 1298 | 9.94 | 23221.46 | Chr06 |
| evm.model.Contig132.4 | OfMYB1R67 | 3456320 | 3458947 | 2627 | 6.61 | 44414.95 | Chr07 |
| evm.model.Contig334.36 | OfMYB1R68 | 3901805 | 3904462 | 2657 | 6.71 | 45630.34 | Chr07 |
| evm.model.Contig275.73 | OfMYB1R69 | 4751946 | 4759356 | 7410 | 6.47 | 73337.37 | Chr07 |
| evm.model.Contig275.45 | OfMYB1R70 | 5081957 | 5086996 | 5039 | 8.44 | 31218.22 | Chr07 |
| evm.model.Contig303.69 | OfMYB1R71 | 5811460 | 5813034 | 1574 | 9.5 | 26734.89 | Chr07 |
| evm.model.Contig81.246 | OfMYB1R72 | 12222167 | 12223105 | 938 | 6.26 | 33646.5 | Chr07 |
| evm.model.Contig160.82 | OfMYB1R73 | 19303417 | 19306445 | 3028 | 5.4 | 33474.67 | Chr07 |
| evm.model.Contig160.18 | OfMYB1R74 | 20130974 | 20134210 | 3236 | 6.28 | 33414.45 | Chr07 |
| evm.model.Contig288.46 | OfMYB1R75 | 23896710 | 23897821 | 1111 | 5.62 | 19977.31 | Chr07 |
| evm.model.Contig116.187 | OfMYB1R76 | 1788743 | 1790893 | 2150 | 7.72 | 46596.15 | Chr08 |
| evm.model.Contig173.62 | OfMYB1R77 | 2506783 | 2516162 | 9379 | 8.43 | 59555.93 | Chr08 |
| evm.model.Contig204.16 | OfMYB1R78 | 3423719 | 3429885 | 6166 | 6.04 | 88998.92 | Chr08 |
| evm.model.Contig405.53 | OfMYB1R79 | 9672444 | 9673328 | 884 | 6.39 | 32125.03 | Chr08 |
| evm.model.Contig361.25 | OfMYB1R80 | 10806421 | 10807305 | 884 | 6.51 | 32180.11 | Chr08 |
| evm.model.Contig133.57 | OfMYB1R81 | 13664063 | 13678813 | 14750 | 5.4 | 82925.12 | Chr08 |
| evm.model.Contig133.81 | OfMYB1R82 | 14125574 | 14127321 | 1747 | 8.78 | 43127.95 | Chr08 |
| evm.model.Contig412.16 | OfMYB1R83 | 14475011 | 14476758 | 1747 | 8.65 | 43111.81 | Chr08 |
| evm.model.Contig32.13 | OfMYB1R84 | 18304548 | 18305987 | 1439 | 8.83 | 29482.09 | Chr08 |
| evm.model.Contig98.90 | OfMYB1R85 | 18901157 | 18903973 | 2816 | 8.87 | 33311.56 | Chr08 |
| evm.model.Contig285.46 | OfMYB1R86 | 27348709 | 27353600 | 4891 | 6.21 | 19759.2 | Chr08 |
| evm.model.Contig467.2 | OfMYB1R87 | 27962647 | 27964008 | 1361 | 8.85 | 35699.23 | Chr08 |
| evm.model.Contig467.20 | OfMYB1R88 | 28205152 | 28209106 | 3954 | 8.93 | 37285.93 | Chr08 |
| evm.model.Contig584.10 | OfMYB1R89 | 29193816 | 29195178 | 1362 | 8.84 | 35716.21 | Chr08 |
| evm.model.Contig628.5 | OfMYB1R90 | 32623153 | 32626891 | 3738 | 6.68 | 127922.4 | Chr08 |
| evm.model.Contig355.2 | OfMYB1R91 | 6391509 | 6400023 | 8514 | 6.01 | 192018.5 | Chr09 |
| evm.model.Contig330.61 | OfMYB1R92 | 10543889 | 10548350 | 4461 | 8.81 | 29757.99 | Chr09 |
| evm.model.Contig59.131 | OfMYB1R93 | 12543403 | 12547679 | 4276 | 5.2 | 52950.59 | Chr09 |
| evm.model.Contig265.50 | OfMYB1R94 | 16270459 | 16273256 | 2797 | 6.76 | 32749.98 | Chr09 |
| evm.model.Contig265.78 | OfMYB1R95 | 16540140 | 16544030 | 3890 | 8.46 | 31520.88 | Chr09 |
| evm.model.Contig339.15 | OfMYB1R96 | 17433377 | 17434942 | 1565 | 8.99 | 42101.74 | Chr09 |
| evm.model.Contig104.17 | OfMYB1R97 | 17849919 | 17851484 | 1565 | 9.09 | 42128.81 | Chr09 |
| evm.model.Contig104.103 | OfMYB1R98 | 18945108 | 18946563 | 1455 | 9.15 | 32038.99 | Chr09 |
| evm.model.Contig542.14 | OfMYB1R99 | 19668453 | 19669908 | 1455 | 9.15 | 32051.05 | Chr09 |
| evm.model.Contig63.101 | OfMYB1R100 | 30006813 | 30008695 | 1882 | 9.1 | 40342.35 | Chr09 |
| evm.model.Contig26.281 | OfMYB1R101 | 3321535 | 3326360 | 4825 | 6.8 | 34317.79 | Chr10 |
| evm.model.Contig26.280 | OfMYB1R102 | 3333307 | 3336310 | 3003 | 6.87 | 36760.29 | Chr10 |
| evm.model.Contig12.103 | OfMYB1R103 | 8924917 | 8927359 | 2442 | 5.99 | 25367.27 | Chr10 |
| evm.model.Contig12.267 | OfMYB1R104 | 10584679 | 10585497 | 818 | 9.63 | 27860.48 | Chr10 |
| evm.model.Contig36.166 | OfMYB1R105 | 14970454 | 14988465 | 18011 | 6.62 | 53151.49 | Chr10 |
| evm.model.Contig21.183 | OfMYB1R106 | 20262390 | 20270066 | 7676 | 5.6 | 44552.09 | Chr10 |
| evm.model.Contig51.99 | OfMYB1R107 | 20453422 | 20468715 | 15293 | 5.6 | 44607.13 | Chr10 |
| evm.model.Contig51.36 | OfMYB1R108 | 21714569 | 21721325 | 6756 | 5.53 | 130026.1 | Chr10 |
| evm.model.Contig123.93 | OfMYB1R109 | 26715412 | 26724561 | 9149 | 7.77 | 30854.77 | Chr10 |
| evm.model.Contig211.94 | OfMYB1R110 | 30999621 | 31000803 | 1182 | 6.42 | 33200.1 | Chr10 |
| evm.model.Contig216.78 | OfMYB1R111 | 31228045 | 31230333 | 2288 | 8.79 | 28173.05 | Chr10 |
| evm.model.Contig216.57 | OfMYB1R112 | 31529166 | 31530369 | 1203 | 9.46 | 28896.86 | Chr10 |
| evm.model.Contig267.49 | OfMYB1R113 | 418464 | 422172 | 3708 | 9.52 | 30024.93 | Chr11 |
| evm.model.Contig395.32 | OfMYB1R114 | 1792805 | 1814625 | 21820 | 6.06 | 84885.27 | Chr11 |
| evm.model.Contig612.2 | OfMYB1R115 | 2374739 | 2377620 | 2881 | 8.72 | 60502.11 | Chr11 |
| evm.model.Contig360.25 | OfMYB1R116 | 2883806 | 2885572 | 1766 | 9.79 | 25698.88 | Chr11 |
| evm.model.Contig327.55 | OfMYB1R117 | 6170612 | 6171008 | 396 | 9.52 | 10537.84 | Chr11 |
| evm.model.Contig327.54 | OfMYB1R118 | 6174514 | 6175392 | 878 | 6.91 | 11460.82 | Chr11 |
| evm.model.Contig136.4 | OfMYB1R119 | 6668602 | 6670591 | 1989 | 5.75 | 45345.76 | Chr11 |
| evm.model.Contig178.5 | OfMYB1R120 | 9609349 | 9611337 | 1988 | 5.68 | 45347.81 | Chr11 |
| evm.model.Contig670.2 | OfMYB1R121 | 11546975 | 11554758 | 7783 | 8.75 | 60020.43 | Chr11 |
| evm.model.Contig256.70 | OfMYB1R122 | 14237729 | 14244233 | 6504 | 9.17 | 37792.92 | Chr11 |
| evm.model.Contig22.153 | OfMYB1R123 | 18622916 | 18623692 | 776 | 9.69 | 15208.32 | Chr11 |
| evm.model.Contig22.155 | OfMYB1R124 | 18645778 | 18646585 | 807 | 8.79 | 16871.06 | Chr11 |
| evm.model.Contig22.158 | OfMYB1R125 | 18678520 | 18679257 | 737 | 5.62 | 9213.17 | Chr11 |
| evm.model.Contig22.159 | OfMYB1R126 | 18705947 | 18706255 | 308 | 6.82 | 11311.48 | Chr11 |
| evm.model.Contig22.197 | OfMYB1R127 | 19138662 | 19140864 | 2202 | 8.53 | 45986.94 | Chr11 |
| evm.model.Contig83.47 | OfMYB1R128 | 5600686 | 5601907 | 1221 | 9.21 | 43747.42 | Chr12 |
| evm.model.Contig230.110 | OfMYB1R129 | 9580651 | 9583346 | 2695 | 6.97 | 32239.24 | Chr12 |
| evm.model.Contig230.52 | OfMYB1R130 | 10116902 | 10117354 | 452 | 7.93 | 13535.29 | Chr12 |
| evm.model.Contig230.50 | OfMYB1R131 | 10133911 | 10134198 | 287 | 9.05 | 10873.99 | Chr12 |
| evm.model.Contig158.13 | OfMYB1R132 | 10831623 | 10832072 | 449 | 6.83 | 13492.26 | Chr12 |
| evm.model.Contig158.14 | OfMYB1R133 | 10838025 | 10838312 | 287 | 9.05 | 10873.99 | Chr12 |
| evm.model.Contig89.28 | OfMYB1R134 | 15365872 | 15371283 | 5411 | 5.93 | 71413.35 | Chr12 |
| evm.model.Contig103.17 | OfMYB1R135 | 27120067 | 27122475 | 2408 | 7.12 | 32508.44 | Chr12 |
| evm.model.Contig407.19 | OfMYB1R136 | 6032918 | 6035099 | 2181 | 6.48 | 41986.34 | Chr13 |
| evm.model.Contig157.11 | OfMYB1R137 | 7599515 | 7600409 | 894 | 5.68 | 20600.08 | Chr13 |
| evm.model.Contig530.31 | OfMYB1R138 | 7807873 | 7808767 | 894 | 5.89 | 20685.23 | Chr13 |
| evm.model.Contig8.258 | OfMYB1R139 | 16035533 | 16038114 | 2581 | 6.48 | 49157.61 | Chr13 |
| evm.model.Contig8.132 | OfMYB1R140 | 17712413 | 17721080 | 8667 | 7.78 | 30730.52 | Chr13 |
| evm.model.Contig10.435 | OfMYB1R141 | 20139489 | 20142252 | 2763 | 6.37 | 44827.23 | Chr13 |
| evm.model.Contig10.369 | OfMYB1R142 | 20838476 | 20844590 | 6114 | 7.49 | 71981.58 | Chr13 |
| evm.model.Contig10.242 | OfMYB1R143 | 22042607 | 22044179 | 1572 | 8.68 | 30452.95 | Chr13 |
| evm.model.Contig130.46 | OfMYB1R144 | 26851351 | 26854341 | 2990 | 5.5 | 62896.75 | Chr13 |
| evm.model.Contig258.94 | OfMYB1R145 | 27519409 | 27524472 | 5063 | 5.89 | 76249.55 | Chr13 |
| evm.model.Contig580.12 | OfMYB1R146 | 28424402 | 28425334 | 932 | 6.29 | 33524.36 | Chr13 |
| evm.model.Contig110.82 | OfMYB1R147 | 1258274 | 1261823 | 3549 | 6.23 | 45432.69 | Chr14 |
| evm.model.Contig151.103 | OfMYB1R148 | 3351119 | 3352283 | 1164 | 9.33 | 35124.36 | Chr14 |
| evm.model.Contig151.30 | OfMYB1R149 | 4107230 | 4112321 | 5091 | 8.91 | 77754.94 | Chr14 |
| evm.model.Contig151.24 | OfMYB1R150 | 4196030 | 4197079 | 1049 | 5.96 | 38356.84 | Chr14 |
| evm.model.Contig99.138 | OfMYB1R151 | 7278764 | 7282193 | 3429 | 5.8 | 74853.04 | Chr14 |
| evm.model.Contig343.14 | OfMYB1R152 | 9249150 | 9254659 | 5509 | 6.11 | 31959.25 | Chr14 |
| evm.model.Contig242.73 | OfMYB1R153 | 13988676 | 13990262 | 1586 | 9.08 | 25556.74 | Chr14 |
| evm.model.Contig150.28 | OfMYB1R154 | 22087317 | 22092594 | 5277 | 9.81 | 28118.95 | Chr14 |
| evm.model.Contig383.20 | OfMYB1R155 | 24609341 | 24612456 | 3115 | 6.01 | 34713.68 | Chr14 |
| evm.model.Contig225.43 | OfMYB1R156 | 29714975 | 29717738 | 2763 | 9.51 | 25790.2 | Chr14 |
| evm.model.Contig19.95 | OfMYB1R157 | 1721464 | 1723749 | 2285 | 9.17 | 28896.24 | Chr15 |
| evm.model.Contig19.321 | OfMYB1R158 | 4087025 | 4089909 | 2884 | 9.1 | 32006.82 | Chr15 |
| evm.model.Contig453.18 | OfMYB1R159 | 5500694 | 5506702 | 6008 | 9.58 | 31407.95 | Chr15 |
| evm.model.Contig453.28 | OfMYB1R160 | 5656110 | 5663186 | 7076 | 6.17 | 68207.52 | Chr15 |
| evm.model.Contig3.27 | OfMYB1R161 | 15007906 | 15008763 | 857 | 9.3 | 10379.86 | Chr15 |
| evm.model.Contig3.202 | OfMYB1R162 | 17599597 | 17604223 | 4626 | 9 | 35616.61 | Chr15 |
| evm.model.Contig3.243 | OfMYB1R163 | 18286746 | 18289389 | 2643 | 6.26 | 52036.14 | Chr15 |
| evm.model.Contig85.158 | OfMYB1R164 | 25102921 | 25108206 | 5285 | 7.59 | 35093.11 | Chr15 |
| evm.model.Contig326.78 | OfMYB1R165 | 26216903 | 26218212 | 1309 | 5.84 | 35170.59 | Chr15 |
| evm.model.Contig144.13 | OfMYB1R166 | 27707568 | 27708497 | 929 | 9.36 | 24150.22 | Chr15 |
| evm.model.Contig353.19 | OfMYB1R167 | 29764938 | 29766531 | 1593 | 9.18 | 24202.19 | Chr15 |
| evm.model.Contig58.202 | OfMYB1R168 | 10676624 | 10677092 | 468 | 9.74 | 13955.62 | Chr16 |
| evm.model.Contig338.27 | OfMYB1R169 | 12978563 | 12995253 | 16690 | 7.88 | 111697.4 | Chr16 |
| evm.model.Contig179.42 | OfMYB1R170 | 15056729 | 15057004 | 275 | 5.63 | 9850.78 | Chr16 |
| evm.model.Contig490.61 | OfMYB1R171 | 15540197 | 15542291 | 2094 | 6.29 | 45435.05 | Chr16 |
| evm.model.Contig169.51 | OfMYB1R172 | 24381514 | 24391861 | 10347 | 9.02 | 42776.84 | Chr16 |
| evm.model.Contig169.106 | OfMYB1R173 | 25105973 | 25113632 | 7659 | 6.42 | 90741.24 | Chr16 |
| evm.model.Contig121.48 | OfMYB1R174 | 4667124 | 4670443 | 3319 | 6.87 | 36803.89 | Chr17 |
| evm.model.Contig393.6 | OfMYB1R175 | 19121787 | 19125431 | 3644 | 5.85 | 33137.55 | Chr17 |
| evm.model.Contig25.220 | OfMYB1R176 | 21089358 | 21093436 | 4078 | 5.75 | 74749.1 | Chr17 |
| evm.model.Contig492.35 | OfMYB1R177 | 24584815 | 24585849 | 1034 | 6.24 | 38278.8 | Chr17 |
| evm.model.Contig492.45 | OfMYB1R178 | 24656449 | 24660016 | 3567 | 9.06 | 77157.46 | Chr17 |
| evm.model.Contig73.241 | OfMYB1R179 | 24686115 | 24689681 | 3566 | 9.01 | 77232.57 | Chr17 |
| evm.model.Contig73.128 | OfMYB1R180 | 25732870 | 25734028 | 1158 | 9.05 | 34358.34 | Chr17 |
| evm.model.Contig79.161 | OfMYB1R181 | 27690632 | 27695250 | 4618 | 5.53 | 57375.19 | Chr17 |
| evm.model.Contig14.191 | OfMYB1R182 | 2187128 | 2189976 | 2848 | 6.52 | 30763.73 | Chr18 |
| evm.model.Contig5.313 | OfMYB1R183 | 6071080 | 6072000 | 920 | 6.25 | 32896.87 | Chr18 |
| evm.model.Contig5.204 | OfMYB1R184 | 7110225 | 7115547 | 5322 | 5.52 | 74498.24 | Chr18 |
| evm.model.Contig5.42 | OfMYB1R185 | 8762389 | 8764290 | 1901 | 9.27 | 29318.1 | Chr18 |
| evm.model.Contig452.21 | OfMYB1R186 | 9295035 | 9298658 | 3623 | 4.49 | 61175.16 | Chr18 |
| evm.model.Contig240.39 | OfMYB1R187 | 10150233 | 10155522 | 5289 | 5.14 | 151307 | Chr18 |
| evm.model.Contig2.41 | OfMYB1R188 | 18937906 | 18938891 | 985 | 7.86 | 31888.95 | Chr18 |
| evm.model.Contig2.63 | OfMYB1R189 | 19698240 | 19709607 | 11367 | 7.19 | 119701.7 | Chr18 |
| evm.model.Contig30.27 | OfMYB1R190 | 25480670 | 25482438 | 1768 | 8.17 | 29857.49 | Chr18 |
| evm.model.Contig30.28 | OfMYB1R191 | 25489345 | 25492299 | 2954 | 6.1 | 40520.99 | Chr18 |
| evm.model.Contig13.168 | OfMYB1R192 | 5623686 | 5625819 | 2133 | 9.07 | 40043.29 | Chr19 |
| evm.model.Contig13.152 | OfMYB1R193 | 5767479 | 5772516 | 5037 | 6.93 | 35906.41 | Chr19 |
| evm.model.Contig197.70 | OfMYB1R194 | 10061586 | 10064645 | 3059 | 8.93 | 42320.32 | Chr19 |
| evm.model.Contig494.19 | OfMYB1R195 | 6760180 | 6775311 | 15131 | 8.38 | 55481.24 | Chr20 |
| evm.model.Contig468.5 | OfMYB1R196 | 6942906 | 6958515 | 15609 | 8.62 | 55564.32 | Chr20 |
| evm.model.Contig279.69 | OfMYB1R197 | 15213029 | 15215161 | 2132 | 7.27 | 42072.6 | Chr20 |
| evm.model.Contig101.22 | OfMYB1R198 | 17180440 | 17181586 | 1146 | 6.45 | 23718.85 | Chr20 |
| evm.model.Contig44.46 | OfMYB1R199 | 18039979 | 18042156 | 2177 | 9.66 | 34244.62 | Chr20 |
| evm.model.Contig44.227 | OfMYB1R200 | 19876481 | 19880323 | 3842 | 5.96 | 69263.56 | Chr20 |
| evm.model.Contig18.78 | OfMYB1R201 | 22099522 | 22104224 | 4702 | 7.79 | 39646.28 | Chr20 |
| evm.model.Contig27.157 | OfMYB1R202 | 1490658 | 1495798 | 5140 | 6.18 | 29763.81 | Chr21 |
| evm.model.Contig212.40 | OfMYB1R203 | 9651010 | 9701057 | 50047 | 8.5 | 40053.46 | Chr21 |
| evm.model.Contig335.62 | OfMYB1R204 | 11181099 | 11193296 | 12197 | 6.35 | 40663.25 | Chr21 |
| evm.model.Contig67.72 | OfMYB1R205 | 24163388 | 24166019 | 2631 | 8.54 | 52993.16 | Chr21 |
| evm.model.Contig16.211 | OfMYB1R206 | 11724335 | 11724779 | 444 | 8.96 | 12734.21 | Chr22 |
| evm.model.Contig16.208 | OfMYB1R207 | 11748547 | 11748852 | 305 | 9.61 | 11734.18 | Chr22 |
| evm.model.Contig16.202 | OfMYB1R208 | 11812807 | 11813320 | 513 | 6.73 | 10843.1 | Chr22 |
| evm.model.Contig7.166 | OfMYB1R209 | 16220597 | 16222843 | 2246 | 7.53 | 85199.31 | Chr22 |
| evm.model.Contig7.257 | OfMYB1R210 | 17424240 | 17440397 | 16157 | 8.15 | 66678.06 | Chr22 |
| evm.model.Contig1.161 | OfMYB1R211 | 10883084 | 10884267 | 1183 | 5.9 | 11141.33 | Chr23 |
| evm.model.Contig1.291 | OfMYB1R212 | 12909033 | 12913318 | 4285 | 8.92 | 31665 | Chr23 |
