# Supplementary material for: Insights Into the MYB-Related Transcription Factors Involved in Regulating Floral Aroma Synthesis in Sweet Osmanthus
Source: Front Plant Sci. 2022 Mar 9;13:765213. doi: 10.3389/fpls.2022.765213 (PMC8959829; doi:10.3389/fpls.2022.765213)
Supplement: Supplementary file 12 [file Table_3.DOCX]

Estimated Ka/Ks ratios and divergence times of tandemly duplicated OfMYB-related genes

| **Duplicated Gene Pairs** | **Ka** | **Ks** | **Ka/Ks** | **Type of Duplication** | **Type of Selection** |
| --- | --- | --- | --- | --- | --- |
| OfMYB1R8 (Chr01) VS. OfMYB1R9(Chr01) | 0.114733319 | 0.199497099 | 0.575112718 | Tandem | Purifying |
| OfMYB1R53 (Chr04) VS. OfMYB1R54((Chr04) | 0.069680032 | 0.177687969 | 0.392148282 | Tandem | Purifying |
| OfMYB1R123 (Chr11) VS. OfMYB1R124(Chr11) | 0.040676446 | 0.044334235 | 0.917495168 | Tandem | Purifying |
| OfMYB1R124 (Chr11) VS. OfMYB1R125(Chr11) | 0.062476974 | 0.225410376 | 0.277169913 | Tandem | Purifying |
| OfMYB1R125 (Chr11) VS. OfMYB1R126(Chr11) | 0.051180439 | 0.173570557 | 0.29486821 | Tandem | Purifying |
| OfMYB1R190 (Chr18) VS. OfMYB1R191(Chr18) | 0.211068042 | 0.508643689 | 0.414962471 | Tandem | Purifying |
| OfMYB1R206 (Chr22) VS. OfMYB1R207(Chr22) | 0.17518272 | 0.227084943 | 0.771441373 | Tandem | Purifying |
