# Supplementary material for: Insights Into the MYB-Related Transcription Factors Involved in Regulating Floral Aroma Synthesis in Sweet Osmanthus
Source: Front Plant Sci. 2022 Mar 9;13:765213. doi: 10.3389/fpls.2022.765213 (PMC8959829; doi:10.3389/fpls.2022.765213)
Supplement: Supplementary file 13 [file Table_4.DOCX]

The RPKM values of OfMYB1R genes in four tissues of sweet osmanthus

| Gene ID | Rename | Root | Stem | Young leaf | Mature leaf | Bud-eye stage | Full blooming stage | Full fading stage |
| --- | --- | --- | --- | --- | --- | --- | --- | --- |
| evm.model.Contig106.66 | MYB1R1 | 100.11 | 38.95 | 10.07 | 42.82 | 13.25 | 17.68 | 24.37 |
| evm.model.Contig167.116 | MYB1R2 | 3.09 | 6.65 | 6.47 | 1.68 | 2.22 | 1.39 | 1.17 |
| evm.model.Contig60.89 | MYB1R3 | 6.07 | 4.02 | 4.37 | 6.58 | 4.89 | 6.27 | 9.92 |
| evm.model.Contig71.216 | MYB1R4 | 0 | 0.01 | 0 | 0.65 | 0 | 0 | 0.05 |
| evm.model.Contig71.92 | MYB1R5 | 23.72 | 19.42 | 15.99 | 19.03 | 9.08 | 6.87 | 7.55 |
| evm.model.Contig66.106 | MYB1R6 | 0.44 | 0 | 0 | 0 | 0.01 | 0 | 0 |
| evm.model.Contig213.15 | MYB1R8 | 0 | 0 | 0 | 0 | 0.01 | 0 | 0 |
| evm.model.Contig213.17 | MYB1R10 | 3.44 | 0.24 | 0 | 0.14 | 3.39 | 1.58 | 0.93 |
| evm.model.Contig283.19 | MYB1R11 | 4.39 | 3.29 | 2.03 | 3.75 | 55.68 | 4.01 | 2.09 |
| evm.model.Contig41.4 | MYB1R12 | 1.79 | 10.07 | 21.01 | 3.37 | 9.81 | 8.22 | 0.35 |
| evm.model.Contig6.17 | MYB1R13 | 7.19 | 3.92 | 2.02 | 7.51 | 3.28 | 2.58 | 3.39 |
| evm.model.Contig50.105 | MYB1R14 | 1.76 | 1.24 | 0.39 | 0.86 | 31.5 | 24.24 | 0.45 |
| evm.model.Contig28.92 | MYB1R15 | 23.86 | 7.65 | 2.37 | 9.62 | 1.7 | 0.8 | 2.35 |
| evm.model.Contig620.8 | MYB1R17 | 4.35 | 3.75 | 2.44 | 5.51 | 0.6 | 0.38 | 0.57 |
| evm.model.Contig52.46 | MYB1R18 | 0.35 | 0.21 | 0.05 | 0 | 0.17 | 0.21 | 0.23 |
| evm.model.Contig282.18 | MYB1R19 | 11 | 9.4 | 9.2 | 16.17 | 2.83 | 1.53 | 3.39 |
| evm.model.Contig45.169 | MYB1R20 | 7.58 | 10.05 | 4.3 | 5.35 | 1.68 | 3.05 | 1.92 |
| evm.model.Contig196.125 | MYB1R21 | 4.69 | 11.36 | 28.77 | 29.22 | 7.94 | 2.97 | 3.85 |
| evm.model.Contig196.85 | MYB1R22 | 14.57 | 15.24 | 21.85 | 25.45 | 11.24 | 11.59 | 11.9 |
| evm.model.Contig206.46 | MYB1R23 | 30.47 | 8.12 | 1.1 | 0.18 | 0.28 | 0.04 | 0.16 |
| evm.model.Contig129.54 | MYB1R24 | 5.44 | 3.71 | 5.04 | 4.16 | 9.19 | 9.4 | 3.88 |
| evm.model.Contig210.43 | MYB1R25 | 8.34 | 7.76 | 9.14 | 24.04 | 1.34 | 2.33 | 6.37 |
| evm.model.Contig269.47 | MYB1R26 | 32.25 | 4.42 | 6.01 | 24.08 | 9.35 | 20.36 | 15.86 |
| evm.model.Contig186.89 | MYB1R27 | 17.56 | 0.41 | 0.89 | 0.15 | 5.2 | 3.47 | 20.37 |
| evm.model.Contig37.265 | MYB1R28 | 5.36 | 9.64 | 6.12 | 7.85 | 3.86 | 1.8 | 2.95 |
| evm.model.Contig300.97 | MYB1R29 | 0 | 0 | 0.01 | 0 | 0 | 0 | 0.02 |
| evm.model.Contig35.10 | MYB1R30 | 0.01 | 0 | 0.01 | 0 | 0 | 0 | 0 |
| evm.model.Contig35.91 | MYB1R31 | 0.82 | 0 | 3.96 | 0.56 | 0 | 0 | 0 |
| evm.model.Contig35.183 | MYB1R32 | 0.23 | 0.03 | 0.02 | 0.38 | 0.1 | 0.22 | 0.2 |
| evm.model.Contig35.291 | MYB1R33 | 0.35 | 0 | 0.01 | 1.52 | 0.21 | 0.36 | 0.51 |
| evm.model.Contig64.205 | MYB1R34 | 5.45 | 7.57 | 11.02 | 12.98 | 11.8 | 12.21 | 8.76 |
| evm.model.Contig64.47 | MYB1R35 | 13.48 | 8.6 | 5.31 | 1.46 | 0.82 | 0.64 | 0.32 |
| evm.model.Contig222.52 | MYB1R37 | 4.98 | 2.92 | 0.15 | 1.84 | 0.16 | 0.15 | 0.1 |
| evm.model.Contig48.24 | MYB1R38 | 10.99 | 2.31 | 0.02 | 1.23 | 0.17 | 0.17 | 11.93 |
| evm.model.Contig131.46 | MYB1R39 | 8.85 | 0.39 | 0.15 | 8.07 | 0.43 | 0.61 | 0.05 |
| evm.model.Contig87.37 | MYB1R41 | 12.34 | 10.33 | 10.58 | 13.5 | 5.02 | 5.03 | 6.46 |
| evm.model.Contig543.11 | MYB1R42 | 2.27 | 2.29 | 3.66 | 1.62 | 1.85 | 0.16 | 0.3 |
| evm.model.Contig624.9 | MYB1R43 | 1.81 | 0.27 | 0.47 | 0.01 | 0.64 | 0.21 | 0.37 |
| evm.model.Contig33.258 | MYB1R45 | 28.5 | 24.7 | 27.19 | 30.81 | 18.76 | 21.3 | 20.37 |
| evm.model.Contig138.55 | MYB1R46 | 14.71 | 10.53 | 10.26 | 15.18 | 13.9 | 21.1 | 21.08 |
| evm.model.Contig328.41 | MYB1R47 | 4.09 | 4.11 | 7.92 | 3.81 | 20.59 | 8.11 | 2.43 |
| evm.model.Contig94.84 | MYB1R48 | 0.14 | 0.36 | 1.04 | 0 | 0 | 0 | 0 |
| evm.model.Contig94.115 | MYB1R49 | 26.07 | 8.25 | 6.3 | 5.96 | 3.55 | 4.8 | 28.05 |
| evm.model.Contig437.42 | MYB1R50 | 33.84 | 0 | 0.09 | 0.65 | 0 | 0 | 0.01 |
| evm.model.Contig305.36 | MYB1R51 | 2.94 | 1.91 | 1.22 | 2.45 | 1.44 | 1.7 | 1.02 |
| evm.model.Contig205.56 | MYB1R52 | 1.78 | 1.82 | 1.32 | 0.84 | 0.21 | 0.19 | 0.12 |
| evm.model.Contig69.76 | MYB1R53 | 0 | 0 | 0 | 0 | 0.68 | 0 | 0 |
| evm.model.Contig69.77 | MYB1R54 | 0 | 0 | 0 | 0 | 0.98 | 0.06 | 0.05 |
| evm.model.Contig273.57 | MYB1R55 | 0.56 | 3.51 | 0 | 3.93 | 0.04 | 0.12 | 4.29 |
| evm.model.Contig221.24 | MYB1R56 | 3.62 | 3.42 | 3.34 | 6.09 | 2.11 | 3.35 | 4.69 |
| evm.model.Contig272.17 | MYB1R57 | 10.22 | 0.47 | 6.91 | 8.94 | 10.4 | 11.48 | 63.55 |
| evm.model.Contig434.4 | MYB1R58 | 1.55 | 0.01 | 0.18 | 0.01 | 0 | 0 | 0 |
| evm.model.Contig227.70 | MYB1R59 | 11.74 | 8.42 | 6.55 | 11.8 | 15 | 17.31 | 14.19 |
| evm.model.Contig170.1 | MYB1R60 | 0 | 0 | 0 | 0 | 2.25 | 1.65 | 2.35 |
| evm.model.Contig78.139 | MYB1R61 | 33.3 | 24.47 | 32.33 | 86.13 | 38.03 | 32.12 | 32.78 |
| evm.model.Contig86.69 | MYB1R62 | 7.41 | 3.14 | 1.17 | 2.47 | 3.55 | 5.95 | 22.44 |
| evm.model.Contig235.33 | MYB1R63 | 74.58 | 5.58 | 1.95 | 0.37 | 10.39 | 21.76 | 5.33 |
| evm.model.Contig296.15 | MYB1R64 | 1.34 | 0 | 0.23 | 0.04 | 0.01 | 0.01 | 0 |
| evm.model.Contig112.70 | MYB1R65 | 8.51 | 8.08 | 7.95 | 11.11 | 6.54 | 7.25 | 8.18 |
| evm.model.Contig388.50 | MYB1R66 | 3.19 | 0.34 | 0.35 | 0.05 | 0 | 0 | 1.81 |
| evm.model.Contig132.4 | MYB1R67 | 4.87 | 5.7 | 1.85 | 10.21 | 0.68 | 0.38 | 0.72 |
| evm.model.Contig334.36 | MYB1R68 | 0.71 | 0.89 | 0.41 | 1.77 | 0.15 | 0.07 | 0.15 |
| evm.model.Contig275.73 | MYB1R69 | 11.96 | 12.77 | 16.28 | 8.66 | 7.23 | 5.22 | 5.64 |
| evm.model.Contig275.45 | MYB1R70 | 1.83 | 1.9 | 1.86 | 4.34 | 127.03 | 240.68 | 0.76 |
| evm.model.Contig303.69 | MYB1R71 | 1.66 | 2 | 2.35 | 0.08 | 0.29 | 0.15 | 0.13 |
| evm.model.Contig81.246 | MYB1R72 | 8.08 | 5.25 | 5.2 | 7.58 | 1.77 | 0.71 | 0.99 |
| evm.model.Contig160.82 | MYB1R73 | 0.01 | 0 | 0 | 0 | 0.15 | 0 | 0 |
| evm.model.Contig160.18 | MYB1R74 | 0 | 0.01 | 0 | 0 | 0 | 0 | 0 |
| evm.model.Contig288.46 | MYB1R75 | 4.05 | 3.69 | 4.05 | 2.5 | 2.46 | 2.74 | 2.98 |
| evm.model.Contig116.187 | MYB1R76 | 10.35 | 9.15 | 1.95 | 7.37 | 1.25 | 1.17 | 0.88 |
| evm.model.Contig173.62 | MYB1R77 | 3.38 | 2.13 | 2.66 | 4.41 | 1.25 | 1.77 | 2.34 |
| evm.model.Contig204.16 | MYB1R78 | 13.08 | 8.23 | 9.29 | 12.12 | 7.66 | 8.02 | 7.86 |
| evm.model.Contig405.53 | MYB1R79 | 1.57 | 1.6 | 1.33 | 3.42 | 0.95 | 1 | 1.26 |
| evm.model.Contig361.25 | MYB1R80 | 1.57 | 1.69 | 1.6 | 2.85 | 0.81 | 0.43 | 0.95 |
| evm.model.Contig133.57 | MYB1R81 | 11.95 | 9.42 | 6.95 | 10.03 | 5.28 | 5.32 | 7.48 |
| evm.model.Contig133.81 | MYB1R82 | 9.34 | 2.03 | 0.42 | 1.37 | 0.86 | 0.43 | 0.87 |
| evm.model.Contig412.16 | MYB1R83 | 4.17 | 2.15 | 0.44 | 2.03 | 0.52 | 0.28 | 0.42 |
| evm.model.Contig32.13 | MYB1R84 | 0.23 | 0.71 | 39.33 | 93.19 | 0 | 0 | 0 |
| evm.model.Contig98.90 | MYB1R85 | 0.24 | 0.53 | 36.21 | 85.72 | 0 | 0 | 0 |
| evm.model.Contig285.46 | MYB1R86 | 10.11 | 20.1 | 16.71 | 19.53 | 8.47 | 4.21 | 2.09 |
| evm.model.Contig467.2 | MYB1R87 | 7.32 | 0.05 | 0 | 0.09 | 0 | 0 | 4.75 |
| evm.model.Contig467.20 | MYB1R88 | 7.15 | 0.86 | 0.72 | 1.34 | 0.46 | 0.73 | 1.11 |
| evm.model.Contig584.10 | MYB1R89 | 9.25 | 0 | 0.05 | 0.1 | 0 | 0 | 0 |
| evm.model.Contig628.5 | MYB1R90 | 1.96 | 0.91 | 0.35 | 2.69 | 0.07 | 0.14 | 0.58 |
| evm.model.Contig355.2 | MYB1R91 | 8.7 | 0.33 | 0.22 | 0 | 1.82 | 0.37 | 0.22 |
| evm.model.Contig330.61 | MYB1R92 | 7.5 | 9.37 | 6.55 | 6.95 | 4 | 3.26 | 5.09 |
| evm.model.Contig59.131 | MYB1R93 | 0 | 0 | 0 | 0 | 0 | 0 | 0 |
| evm.model.Contig265.50 | MYB1R94 | 0.21 | 1.01 | 1.19 | 0 | 0 | 0 | 0.03 |
| evm.model.Contig265.78 | MYB1R95 | 3.61 | 4.1 | 2.86 | 10.33 | 1.62 | 0.46 | 1.73 |
| evm.model.Contig339.15 | MYB1R96 | 3.94 | 0.04 | 0.35 | 0.14 | 0 | 0 | 0 |
| evm.model.Contig104.17 | MYB1R97 | 5.23 | 0.08 | 0.36 | 0.22 | 0 | 0 | 0 |
| evm.model.Contig104.103 | MYB1R98 | 5.09 | 4.57 | 1.78 | 4.65 | 2.38 | 0.55 | 0.75 |
| evm.model.Contig542.14 | MYB1R99 | 6.8 | 4.65 | 1.64 | 3.82 | 1.1 | 0.33 | 0.48 |
| evm.model.Contig63.101 | MYB1R100 | 1.72 | 4.63 | 1.03 | 0.14 | 0.22 | 0 | 0.28 |
| evm.model.Contig12.103 | MYB1R103 | 12.26 | 3.54 | 1.08 | 4.34 | 4.11 | 9.19 | 37.44 |
| evm.model.Contig12.267 | MYB1R104 | 22.51 | 8.9 | 0.66 | 1.95 | 0.59 | 0.73 | 0.49 |
| evm.model.Contig36.166 | MYB1R105 | 0.35 | 0.26 | 0.14 | 0.2 | 0.26 | 0.58 | 0.42 |
| evm.model.Contig21.183 | MYB1R106 | 14.04 | 11.49 | 16.3 | 13.53 | 13 | 20.23 | 26.11 |
| evm.model.Contig51.99 | MYB1R107 | 8.43 | 6.87 | 8.79 | 9.99 | 8.72 | 9.45 | 15.21 |
| evm.model.Contig51.36 | MYB1R108 | 15.83 | 14.94 | 17.05 | 18.7 | 10.03 | 7.94 | 10.89 |
| evm.model.Contig123.93 | MYB1R109 | 7.99 | 10.79 | 11.68 | 8.99 | 8.32 | 2.74 | 2.19 |
| evm.model.Contig211.94 | MYB1R110 | 10.98 | 11.58 | 12.77 | 6.06 | 52.18 | 9.47 | 4.44 |
| evm.model.Contig216.78 | MYB1R111 | 0.76 | 0.18 | 0 | 0.4 | 0.1 | 0.13 | 0.12 |
| evm.model.Contig216.57 | MYB1R112 | 11.02 | 0 | 0 | 0.36 | 0 | 0 | 0 |
| evm.model.Contig267.49 | MYB1R113 | 0.26 | 0.53 | 0.48 | 0.32 | 0.35 | 0.48 | 2.48 |
| evm.model.Contig395.32 | MYB1R114 | 14.45 | 13.65 | 11.73 | 27.03 | 158.31 | 266.47 | 6.94 |
| evm.model.Contig612.2 | MYB1R115 | 0.31 | 0.45 | 0.81 | 0.24 | 0.16 | 0.16 | 0.35 |
| evm.model.Contig360.25 | MYB1R116 | 2.16 | 0 | 0 | 0 | 0 | 0 | 0.03 |
| evm.model.Contig327.55 | MYB1R117 | 0.77 | 3.33 | 0.6 | 0 | 0.7 | 0.29 | 0.11 |
| evm.model.Contig327.54 | MYB1R118 | 0.07 | 0.04 | 0.57 | 0.46 | 1.18 | 3.11 | 0.17 |
| evm.model.Contig136.4 | MYB1R119 | 16.28 | 15.27 | 2.89 | 13.33 | 2.03 | 1.18 | 3.07 |
| evm.model.Contig178.5 | MYB1R120 | 12.31 | 16.52 | 2.79 | 10.61 | 2.21 | 0.8 | 2.2 |
| evm.model.Contig670.2 | MYB1R121 | 0.02 | 0.08 | 0.34 | 0.03 | 0.01 | 0 | 0 |
| evm.model.Contig256.70 | MYB1R122 | 12.24 | 12.3 | 11.03 | 11.54 | 19.7 | 28.87 | 10.05 |
| evm.model.Contig22.153 | MYB1R123 | 0.02 | 0.02 | 0.02 | 0.1 | 0 | 0.02 | 0.02 |
| evm.model.Contig22.158 | MYB1R125 | 0 | 0 | 0 | 6.17 | 0.03 | 0 | 0.08 |
| evm.model.Contig22.159 | MYB1R126 | 0.04 | 0 | 0 | 2.12 | 0 | 0 | 0 |
| evm.model.Contig22.197 | MYB1R127 | 11.51 | 21.75 | 5.32 | 3.53 | 4.72 | 1.53 | 0.73 |
| evm.model.Contig230.110 | MYB1R129 | 1.18 | 0.24 | 9.21 | 30.18 | 0 | 0 | 0.02 |
| evm.model.Contig230.52 | MYB1R130 | 0.06 | 0.93 | 0.32 | 0.02 | 0.63 | 0.07 | 0 |
| evm.model.Contig230.50 | MYB1R131 | 0.14 | 0.51 | 5.83 | 3.72 | 0.65 | 0.18 | 0 |
| evm.model.Contig158.13 | MYB1R132 | 0.04 | 0 | 0 | 0 | 0 | 0 | 0.05 |
| evm.model.Contig158.14 | MYB1R133 | 0.25 | 0.63 | 6.96 | 3.66 | 0.56 | 0.13 | 0.06 |
| evm.model.Contig89.28 | MYB1R134 | 4.66 | 2.51 | 3.37 | 3.95 | 1.22 | 0.32 | 0.23 |
| evm.model.Contig103.17 | MYB1R135 | 0.1 | 0 | 0 | 0 | 0 | 0 | 0 |
| evm.model.Contig407.19 | MYB1R136 | 0.06 | 0.78 | 0.03 | 0.3 | 1 | 0.43 | 0 |
| evm.model.Contig157.11 | MYB1R137 | 0.74 | 0.24 | 0.36 | 0.25 | 0.32 | 0.6 | 0.99 |
| evm.model.Contig530.31 | MYB1R138 | 0.74 | 0.18 | 0.21 | 0.56 | 0.27 | 0.39 | 0.69 |
| evm.model.Contig8.258 | MYB1R139 | 4.48 | 2.4 | 1.99 | 1.62 | 0.9 | 0.17 | 0.5 |
| evm.model.Contig10.435 | MYB1R141 | 5.27 | 4.14 | 2.93 | 7.58 | 1.86 | 1.56 | 1.53 |
| evm.model.Contig10.242 | MYB1R143 | 5.28 | 4.12 | 2.42 | 3.68 | 2.48 | 1.52 | 0.69 |
| evm.model.Contig130.46 | MYB1R144 | 0.05 | 0.19 | 0.01 | 0 | 0.09 | 0.01 | 0.26 |
| evm.model.Contig258.94 | MYB1R145 | 25.37 | 17.88 | 10.67 | 11.06 | 33.65 | 36.24 | 13.17 |
| evm.model.Contig580.12 | MYB1R146 | 3.2 | 4.27 | 7.78 | 6.09 | 2.97 | 1.69 | 1.46 |
| evm.model.Contig151.103 | MYB1R148 | 4.37 | 8.23 | 7.35 | 1.13 | 14.19 | 11.31 | 2.92 |
| evm.model.Contig151.30 | MYB1R149 | 4.14 | 6.1 | 5.2 | 5.43 | 2.3 | 1.11 | 1 |
| evm.model.Contig151.24 | MYB1R150 | 2.58 | 5.11 | 5.17 | 7.83 | 0.35 | 0.42 | 4.57 |
| evm.model.Contig99.138 | MYB1R151 | 11.15 | 5.35 | 4.41 | 5.81 | 17.55 | 23.67 | 9.76 |
| evm.model.Contig343.14 | MYB1R152 | 3.74 | 8.36 | 27.76 | 0.27 | 26.05 | 10.69 | 1.76 |
| evm.model.Contig242.73 | MYB1R153 | 0 | 0.07 | 0 | 0.3 | 0 | 0 | 0.02 |
| evm.model.Contig150.28 | MYB1R154 | 0.08 | 1.46 | 0 | 0 | 0 | 0 | 0 |
| evm.model.Contig383.20 | MYB1R155 | 23.03 | 33.11 | 28.63 | 53.14 | 12 | 13.23 | 45.33 |
| evm.model.Contig225.43 | MYB1R156 | 0 | 1.49 | 0 | 0 | 0 | 0 | 0 |
| evm.model.Contig19.95 | MYB1R157 | 149.65 | 170.28 | 40.97 | 147.72 | 41.63 | 25.05 | 48.31 |
| evm.model.Contig19.321 | MYB1R158 | 0.73 | 0.94 | 1.19 | 0.42 | 1.94 | 4.68 | 6.67 |
| evm.model.Contig3.27 | MYB1R161 | 0.24 | 0.05 | 0.05 | 2.54 | 0.1 | 0.09 | 0.1 |
| evm.model.Contig3.243 | MYB1R163 | 13.12 | 8.21 | 5.01 | 35.87 | 1.29 | 0.57 | 1.69 |
| evm.model.Contig85.158 | MYB1R164 | 15.16 | 12.23 | 10.91 | 12.36 | 7.8 | 4.45 | 6.37 |
| evm.model.Contig326.78 | MYB1R165 | 0.05 | 1.44 | 0.24 | 5.84 | 0.72 | 0.08 | 0.85 |
| evm.model.Contig144.13 | MYB1R166 | 0 | 0 | 0 | 0 | 0.04 | 0 | 0 |
| evm.model.Contig353.19 | MYB1R167 | 3.22 | 1.45 | 2.66 | 2.75 | 19.41 | 35.71 | 2.9 |
| evm.model.Contig58.202 | MYB1R168 | 0.16 | 1.1 | 0.31 | 3.43 | 0.39 | 0.4 | 0.24 |
| evm.model.Contig338.27 | MYB1R169 | 31.26 | 21.67 | 15.65 | 37.13 | 26.16 | 51.47 | 20.95 |
| evm.model.Contig179.42 | MYB1R170 | 0.54 | 0 | 0 | 0 | 1.81 | 0 | 0.1 |
| evm.model.Contig490.61 | MYB1R171 | 32.89 | 25.07 | 2.74 | 29.43 | 0.93 | 0.31 | 0.89 |
| evm.model.Contig169.51 | MYB1R172 | 1.27 | 0 | 0 | 0 | 0 | 0 | 0 |
| evm.model.Contig169.106 | MYB1R173 | 9.05 | 0.85 | 0.34 | 1.49 | 146.61 | 393.92 | 0.97 |
| evm.model.Contig121.48 | MYB1R174 | 2.82 | 2.42 | 2.8 | 4.44 | 2.8 | 3.47 | 2.82 |
| evm.model.Contig393.6 | MYB1R175 | 2.43 | 2.84 | 6.35 | 14.31 | 26.72 | 27.58 | 2.03 |
| evm.model.Contig25.220 | MYB1R176 | 20.08 | 14.41 | 15.5 | 14.06 | 24.64 | 13.27 | 8.19 |
| evm.model.Contig492.35 | MYB1R177 | 1.08 | 5.82 | 4.93 | 8.19 | 0.02 | 0.03 | 2.92 |
| evm.model.Contig492.45 | MYB1R178 | 2.67 | 10.7 | 14.28 | 14.82 | 3.05 | 1.24 | 0.74 |
| evm.model.Contig73.241 | MYB1R179 | 2.76 | 10.29 | 12.92 | 15.48 | 2.69 | 1.27 | 0.8 |
| evm.model.Contig73.128 | MYB1R180 | 6.62 | 4.91 | 2.8 | 7.3 | 17.12 | 29.5 | 16.83 |
| evm.model.Contig79.161 | MYB1R181 | 35.45 | 14.56 | 10.92 | 21.85 | 27.03 | 30.01 | 30.23 |
| evm.model.Contig14.191 | MYB1R182 | 32.34 | 27.46 | 29.16 | 20.97 | 32.43 | 18.09 | 14.1 |
| evm.model.Contig5.313 | MYB1R183 | 8.51 | 12.09 | 12.06 | 5.88 | 4.76 | 3.35 | 5.49 |
| evm.model.Contig5.204 | MYB1R184 | 26.64 | 15.68 | 9.64 | 13.99 | 7.8 | 9.58 | 14.41 |
| evm.model.Contig5.42 | MYB1R185 | 158.6 | 4.15 | 1.93 | 20.57 | 1.46 | 0.87 | 0.6 |
| evm.model.Contig240.39 | MYB1R187 | 24.09 | 20.18 | 16.92 | 25.13 | 15.87 | 14.39 | 19.29 |
| evm.model.Contig2.41 | MYB1R188 | 1.13 | 3.49 | 9.62 | 9.24 | 0.08 | 0.01 | 0.34 |
| evm.model.Contig2.63 | MYB1R189 | 3.03 | 2.9 | 2.85 | 3.2 | 0.72 | 0.49 | 1.33 |
| evm.model.Contig30.27 | MYB1R190 | 0.03 | 0.03 | 0.01 | 0 | 0.01 | 0 | 0.01 |
| evm.model.Contig30.28 | MYB1R191 | 0.03 | 0 | 0 | 0 | 0 | 0 | 0 |
| evm.model.Contig13.168 | MYB1R192 | 4.04 | 6.38 | 3.67 | 4.62 | 1.3 | 1.04 | 1.83 |
| evm.model.Contig13.152 | MYB1R193 | 23.16 | 10.5 | 17.12 | 9.15 | 11 | 19.88 | 7.89 |
| evm.model.Contig197.70 | MYB1R194 | 13.12 | 2.15 | 0.24 | 0.67 | 0.02 | 0 | 0.65 |
| evm.model.Contig494.19 | MYB1R195 | 7.19 | 6.76 | 6.13 | 6.26 | 3.1 | 2.11 | 2.57 |
| evm.model.Contig468.5 | MYB1R196 | 6.15 | 5.92 | 5.84 | 7.23 | 2.6 | 2.48 | 3.01 |
| evm.model.Contig279.69 | MYB1R197 | 6.52 | 2.64 | 9.04 | 6.51 | 15.7 | 7.98 | 7.92 |
| evm.model.Contig101.22 | MYB1R198 | 3.13 | 5.33 | 2.72 | 4.55 | 0.74 | 0.33 | 0.91 |
| evm.model.Contig44.46 | MYB1R199 | 141.19 | 6.29 | 2.28 | 9.75 | 38.21 | 60.09 | 20.07 |
| evm.model.Contig44.227 | MYB1R200 | 4.89 | 2.95 | 2.77 | 3.65 | 36.21 | 2.54 | 1.73 |
| evm.model.Contig18.78 | MYB1R201 | 6.39 | 10.86 | 12.9 | 10.87 | 31.85 | 25.72 | 19.43 |
| evm.model.Contig27.157 | MYB1R202 | 1.99 | 1.26 | 1.41 | 0.51 | 2.45 | 0.91 | 0.2 |
| evm.model.Contig212.40 | MYB1R203 | 54.99 | 3.96 | 4.52 | 3.2 | 9.59 | 10.9 | 15.63 |
| evm.model.Contig335.62 | MYB1R204 | 14.15 | 11.92 | 14.39 | 21.03 | 10.51 | 3.82 | 3.68 |
| evm.model.Contig67.72 | MYB1R205 | 9.33 | 7.05 | 9.28 | 10.02 | 4.61 | 5 | 6.87 |
| evm.model.Contig16.211 | MYB1R206 | 0.29 | 0.31 | 0.22 | 1.69 | 2.11 | 0.39 | 0.07 |
| evm.model.Contig16.208 | MYB1R207 | 0.93 | 4.65 | 2.17 | 1.26 | 3.29 | 1.4 | 0.17 |
| evm.model.Contig16.202 | MYB1R208 | 0.24 | 0 | 0 | 4.49 | 0.96 | 0.56 | 0.05 |
| evm.model.Contig7.166 | MYB1R209 | 12.28 | 9.5 | 8.74 | 8.08 | 5.27 | 3.43 | 6.02 |
| evm.model.Contig7.257 | MYB1R210 | 0.01 | 0.02 | 0.02 | 0.08 | 0.55 | 0.02 | 0 |
| evm.model.Contig1.161 | MYB1R211 | 0 | 0.94 | 0 | 0 | 0.1 | 0 | 0 |
| evm.model.Contig1.291 | MYB1R212 | 10.13 | 10.41 | 11.62 | 18.8 | 6.47 | 7.89 | 9.16 |
