# Supplementary material for: Insights Into the MYB-Related Transcription Factors Involved in Regulating Floral Aroma Synthesis in Sweet Osmanthus
Source: Front Plant Sci. 2022 Mar 9;13:765213. doi: 10.3389/fpls.2022.765213 (PMC8959829; doi:10.3389/fpls.2022.765213)
Supplement: Supplementary file 14 [file Table_5.DOCX]

Identity and quantity of volatile compounds with relative content in the four types of plants with transient expression.

| Cas # | Fragrance molecules | RIL value | Content/(μg/kg) | | | | |
| --- | --- | --- | --- | --- | --- | --- | --- |
|  |  |  | CK | OfMYB1R70 | OfMYB1R114 | OfMYB1R201 | |
| 644-78-0 | 2-Hydroxychalcone |  | 0.0028±0.0003 | 0.0344±0.0071 | 0.1682±0.0577 | | 0.0014±0.0006 |
| 3820-67-5 | Glafenin |  | 0.0208±0.0031 | 0.1943±0.0322 | 0.8826±0.2606 | | 0.0052±0.0014 |
| 122-78-1 | Benzeneacetaldehyde | 1046 | 0.0002±0.0002 | 0.000±0.000 | 0.0293±0.0202 | | 0.000±0.000 |
| 2519-37-1 | Heptanoic acid,6-methyl-, methyl ester | 1505 | 0.0080±0.0024 | 0.0891±0.0181 | 0.5522±0.1865 | | 0.1785±0.0695 |
| 111-11-5 | Octanoic acid, methyl ester | 1513 | 0.0085±0.0021 | 0.1114±0.0221 | 0.5694±0.1713 | | 0.0043±0.0020 |
| 124-19-6 | Nonanal | 1528 | 0.0375±0.00611 | 0.0350±0.0067 | 0.6285±0.2031 | | 0.0011±0.0003 |
| 4971-56-6 | 2,4(3H,5H)-Furandione |  | 0.0029±0.0011 | 0.0173±0.0034 | 0.1179±0.0458 | | 0.0009±0.0002 |
| 24683-00-9 | Pyrazine, 2-methoxy-3-(2-methylpropyl)- | 1609 | 0.0083±0.0021 | 0.0846±0.0151 | 0.4108±0.1513 | | 0.0021±0.0008 |
| 1502-05-2 | Cyclodecanol | 1518 | 0.0106±0.0022 | 0.0107±0.0037 | 0.1696±0.0497 | | 0.0000±0.0000 |
| 432-25-7 | β-cyclocitral | 1537 | 0.0026±0.0003 | 0.0158±0.0050 | 0.1402±0.0583 | | 0.0430±0.0198 |
| 54-11-5 | Pyridine, 3-(1-methyl-2-pyrrolidinyl)-, (S)- | 1665 | 0.0087±0.0039 | 0.0372±0.0108 | 0.1993±0.1446 | | 0.0001±0.0001 |
| 79-77-6 | β-Ionone | 1485 | 0.0019±0.0003 | 0.0189±0.0028 | 0.1453±0.0726 | | 0.0003±0.0002 |
| 23676-09-7 | Benzoic acid, 4-ethoxy-, ethyl ester | 1698 | 0.0029±0.0007 | 0.0173±0.0050 | 0.1107±0.0275 | | 0.0005±0.0002 |
| 6846-50-0 | 2,2,4-trimethyl-1,3-pentanediol diisobutyrate | 1313 | 0.0036±0.0006 | 0.0543±0.0155 | 0.2297±0.0683 | | 0.0014±0.0007 |
| 55124-79-3 | Heptadecane, 9-hexyl- |  | 0.0009±0.0005 | 0.0164±0.0044 | 0.1881±0.0729 | | 0.0007±0.0003 |
| 38472-90-1 | 5,6,7-Trimethoxy-1-indanone | 1694 | 0.0075±0.0014 | 0.0745±0.0204 | 0.4947±0.1347 | | 0.0015±0.0004 |
| 124-10-7 | Methyl tetradecanoate | 1723 | 0.0012±0.0005 | 0.0140±0.0023 | 0.0462±0.0179 | | 0.0012±0.0004 |
| 55724-48-6 | Dasycarpidan-1-methanol, acetate (ester) | 1199 | 0.0013±0.0005 | 0.0395±0.0233 | 0.0747±0.0291 | | 0.0006±0.0002 |
| 112-39-0 | Methyl hexadecanoate | 1923 | 0.0321±0.0040 | 0.3003±0.0704 | 1.0357±0.3609 | | 0.0477±0.0111 |
| 84-74-2 | Dibutyl phthalate | 1639 | 0.0291±0.0078 | 0.1769±0.0364 | 0.8010±0.2111 | | 0.0062±0.0019 |
| 593-49-7 | Hexadecanoic acid, methyl ester |  | 0.0062±0.0038 | 0.0000±0.0000 | 0.0000±0.0000 | | 0.1160±0.0555 |
| 1193-24-4 | 4,6-Dihydroxypyrimidine |  | 0.0008±0.0005 | 0.0123±0.0098 | 0.0572±0.0335 | | 0.0017±0.0009 |
| 5129-61-3 | Methyl16-methylheptadecanoate | 2564 | 0.0213±0.0067 | 0.1394±0.0298 | 0.7000±0.1355 | | 0.0094±0.0032 |
