# Supplementary material for: Insights Into the MYB-Related Transcription Factors Involved in Regulating Floral Aroma Synthesis in Sweet Osmanthus
Source: Front Plant Sci. 2022 Mar 9;13:765213. doi: 10.3389/fpls.2022.765213 (PMC8959829; doi:10.3389/fpls.2022.765213)
Supplement: Supplementary file 16 [file Table_7.docx]

| Name of VOCs | CK | | | | MYB1R70 | | | | | | MYB1R114 | | | | | | MYB1R201 | | | | | |
| --- | --- | --- | --- | --- | --- | --- | --- | --- | --- | --- | --- | --- | --- | --- | --- | --- | --- | --- | --- | --- | --- | --- |
|  | CK-1 | CK-2 | CK-3 | CK-4 | MYB1R70-1 | MYB1R70-2 | MYB1R70-3 | MYB1R70-4 | MYB1R70-5 | MYB1R70-6 | MYB1R114-1 | MYB1R114-2 | MYB1R114-3 | MYB1R114-4 | MYB1R114-5 | MYB1R114-6 | MYB1R201-1 | MYB1R201-2 | MYB1R201-3 | MYB1R201-4 | MYB1R201-5 | MYB1R201-6 |
| 2-Hydroxychalcone | 10270804.53 | 10009078.35 | 9612314.82 | 11130702.46 | 7596326.1 | 9046823 | 17305279 | 17305278.55 | 18126926.38 | 14665989.37 | 25467583.19 | 31638495.02 | 12432830.42 | 11102594.98 | 5641152.39 | 14806497.62 | 17815632.16 | 14165740.78 | 14828454.2 | 6777441.78 | 9154486.34 | 11367238.37 |
| Glafenin | 68676133.6 | 75088435.27 | 82341429.93 | 70935228.38 | 78521113 | 66662643 | 67460469 | 104375376.3 | 83990197.55 | 80966060.26 | 118477879.5 | 54981726.01 | 137715411.2 | 60164770.46 | 53058916.2 | 55156135.36 | 45920419.49 | 17910272.55 | 62444930.95 | 91926465.08 | 55230548.29 | 70164352.72 |
| Phenylacetaldehyde | 0.00001 | 0.00001 | 0.00001 | 3394936.48 | 0.00001 | 0.00001 | 0.00001 | 0.00001 | 0.00001 | 0.00001 | 4822008.97 | 0.00001 | 10891277.15 | 4413082.56 | 0.00001 | 0.00001 | 0.00001 | 0.00001 | 0.00001 | 0.00001 | 0.00001 | 0.00001 |
| Heptanoic acid,6-methyl-, methyl ester | 34875277.97 | 19645474.13 | 11895407.32 | 56591356.46 | 38289613 | 12110660 | 33123946 | 44527558.63 | 70275890.01 | 42816227.9 | 33302888.31 | 20706700.56 | 108351837.2 | 61535070.36 | 19158759.07 | 28453145.77 | 91584827.67 | 255163324.3 | 24329078.07 | 27994121.36 | 44197890.68 | 53011269.62 |
| Methyl octanoate | 33396529.77 | 21359779.89 | 18322587.16 | 55880316.11 | 46832770 | 18645943 | 32828018 | 57842898.29 | 90749753.77 | 56120436.62 | 35006261.64 | 30501626.5 | 75251220.85 | 51381604.44 | 29585188.78 | 39608526.46 | 57823747.28 | 88239577.27 | 18777305.81 | 23222899.64 | 22630790.88 | 32085279.72 |
| Nonanal | 103410867.1 | 151635424.6 | 135895134.4 | 140783890.2 | 33566897 | 5127247.3 | 4946279 | 16350662.41 | 18443266.26 | 27478739.03 | 104568228.9 | 73698733.51 | 135098468.4 | 48065157.29 | 19926642.82 | 28628430.22 | 0.00001 | 10151205.76 | 13139066.63 | 50122663.87 | 11411747.22 | 19837382.87 |
| tetronic acid | 6629056.53 | 10971758.92 | 3595200.72 | 21565812.3 | 5376008.5 | 4309784.6 | 3964335.7 | 10492492.67 | 13409313.78 | 8404698.32 | 24715282.45 | 9012826.62 | 30018263.77 | 9177551.78 | 5695786.13 | 3084005.07 | 5657637.84 | 19868458.34 | 11196822.3 | 0.00001 | 10253017.18 | 11524479.82 |
| 2-Methoxy-3-isobutyl pyrazine | 32420490.14 | 24709433.16 | 14464458.45 | 54426093.85 | 35692058 | 12715662 | 43573526 | 53865645.09 | 44943321.3 | 33920091.01 | 67608940.42 | 25631283.81 | 39719982.76 | 19111131.5 | 28848027.32 | 35828045.61 | 11707995.67 | 0.00001 | 0.00001 | 34683077.99 | 39743092.42 | 75278207.89 |
| 2-Methylnorbornane | 21454638.95 | 46584242.83 | 38681782.37 | 41042580.75 | 7018544.3 | 0.00001 | 0.00001 | 8557295.64 | 8147042.87 | 14054204.04 | 40712207.26 | 13302318.07 | 30906227.44 | 17057025.93 | 6493667.98 | 8532804.12 | 0.00001 | 0.00001 | 0.00001 | 0.00001 | 0.00001 | 0.00001 |
| β-cyclocitral | 8731420.13 | 10513534.88 | 7411170.63 | 10626708.29 | 15515751 | 6248797.8 | 0.00001 | 4656055.32 | 7430616.03 | 7733808.67 | 8970714.26 | 6117587.05 | 11710439.58 | 7438486.54 | 8778354.75 | 13846045.7 | 0.00001 | 0.00001 | 0.00001 | 5749778.57 | 0.00001 | 0.00001 |
| L-Nicotine | 74054840.38 | 52714594.19 | 0.00001 | 16046816.82 | 42898842 | 5228014.7 | 7858973.4 | 0.00001 | 16871627.77 | 36345115.41 | 9705385.35 | 3695948.7 | 7230750.78 | 8269197.16 | 44551901.49 | 2449278.77 | 0.00001 | 10520928.19 | 0.00001 | 0.00001 | 0.00001 | 0.00001 |
| Decanoic acid, ethyl ester | 5120282256 | 3274680296 | 2951449322 | 3769567591 | 549675843 | 201633219 | 294325449 | 721352288.6 | 414071325.7 | 700552843.4 | 12325288900 | 171256621.9 | 91254149.78 | 78456235.7 | 48539707.46 | 34557899.49 | 4096791341 | 16925029960 | 10198390408 | 21357838625 | 12629919835 | 15866395071 |
| β-Ionone | 13608810.58 | 6311408.28 | 5198348.34 | 5284387.47 | 12438848 | 4978793.6 | 5114252.1 | 4451326.05 | 8190271.89 | 16071402.59 | 7981858.97 | 4549837.48 | 7973011.61 | 8617453.99 | 7701308.12 | 16897939.7 | 0.00001 | 0.00001 | 0.00001 | 18447500.66 | 6027870.43 | 10051841.2 |
| Ethyl 4-etoxybenzoate | 8291628.64 | 16018378.53 | 7928375.48 | 8886405.57 | 0.00001 | 7231967.7 | 5702756.3 | 13422234.15 | 9069565.6 | 5550903.69 | 23269540.06 | 21340567.95 | 11043383.57 | 8311250.22 | 10314426.69 | 3385891.09 | 0.00001 | 0.00001 | 8015090.68 | 14156060.94 | 10866998.8 | 12440241.95 |
| 2,2,4-trimethyl-1,3-pentanediol diisobutyrate | 9128499.75 | 14360186.57 | 11856213.01 | 15414532.16 | 14064359 | 22877749 | 14832162 | 25499415.39 | 35320127.44 | 10888591.88 | 28767879.65 | 14856045.28 | 19134612.51 | 20732390.84 | 17934540.99 | 15398825.58 | 20230014.45 | 11698262.92 | 5762054.27 | 15978106.28 | 10866969.55 | 10571578.74 |
| Heneicosane | 0.00001 | 4699691.19 | 6523506.25 | 0.00001 | 12843686 | 6068244.8 | 7172481.6 | 4698967.8 | 3569073.8 | 3635249.86 | 0.00001 | 0.00001 | 18058772.94 | 12328784.17 | 15566058.96 | 15659455.16 | 7735268.49 | 0.00001 | 14337171.1 | 9681998.57 | 5637739.79 | 3037750.24 |
| 2,5-Di-tert-butylhydroquinone | 26268998.12 | 19522162.28 | 33644948.33 | 27683943.42 | 28894897 | 27960413 | 40367367 | 37494485.83 | 16227057.16 | 19220410.35 | 38130831.73 | 39903001.98 | 48784608.43 | 41415575.73 | 40189776.82 | 29091191.77 | 9617645.73 |  | 11083735.92 | 27050120.37 | 27636419.47 | 28791092.34 |
| Tetradecanoic acid, methyl ester | 0.00001 | 7207416.12 | 4211428.8 | 4279669.81 | 6668887.2 | 3576580.2 | 6865494 | 5661580.86 | 5413904.46 | 7030873.27 | 11848401.8 | 4353962.5 | 8475593.58 | 4792017.84 | 4699270.84 | 0.00001 | 4988280.21 | 25912884.04 | 29865732.07 | 17060823.87 | 5055166.32 | 5530826.58 |
| Dimethoxylycopene | 0.00001 | 7169648.42 | 3967121.83 | 5530289.97 | 15856646 | 30229703 | 7289628.9 | 10974065.33 | 5383205.85 | 3765820.27 | 19342116.01 | 4480498.74 | 9065068.17 | 3775605.58 | 3425428.45 | 6997617.04 | 0.00001 | 0.00001 | 15978434.67 | 22725318.61 | 6154703.28 | 10105383.32 |
| Methyl hexadecanoate | 213304110.2 | 114102690.7 | 85913685.11 | 86627105.89 | 258183567 | 111194434 | 72120228 | 112949301 | 105297887.8 | 87162952.92 | 171331662.6 | 100282321.2 | 78774753.21 | 52141244.18 | 75817904.36 | 87247929.82 | 1034993515 | 0.00001 | 198544402.6 | 158275897.7 | 83993340.41 | 0.00001 |
| Dibutyl phthalate | 79462753.54 | 161212632.7 | 98981766.86 | 67648217.03 | 73831214 | 54769573 | 87631609 | 75175649.59 | 71222533.68 | 57165788.28 | 42758267.62 | 24217663.55 | 62802924.6 | 60879717.96 | 52188051.19 | 73318523.88 | 42541134.85 | 79591298.77 | 131236793.5 | 90947489.1 | 60030254.7 | 0.00001 |
| Heptacosane | 4701547.51 | 24290134.03 | 48976823.65 | 0.00001 | 0.00001 | 0.00001 | 0.00001 | 0.00001 | 0.00001 | 0.00001 | 0.00001 | 0.00001 | 0.00001 | 0.00001 | 0.00001 | 0.00001 | 2406003722 | 87604204.83 | 999713905.5 | 67668283.58 | 27094078.67 | 0.00001 |
| 4,6-Dihydroxypyrimidine | 9370283.52 | 0.00001 | 4036596.35 | 0.00001 | 32919100 | 0.00001 | 0.00001 | 0.00001 | 0.00001 | 9643768.19 | 16288572.5 | 2776791.29 | 14449503.17 | 13145126.56 | 0.00001 | 0.00001 | 0.00001 | 0.00001 | 0.00001 | 212680694.1 | 0.00001 | 0.00001 |
| Methyl16-methylheptadecanoate | 31425758.21 | 174743984.7 | 36920906.43 | 49766878.47 | 87472516 | 0.00001 | 81417432 | 103885949 | 80143209.7 | 44077179.75 | 29328791.29 | 13932896.92 | 82967778.97 | 51680892.85 | 51512412.55 | 51382930.5 | 0.00001 | 664280695.9 | 0.00001 | 239833549.9 | 77146361.78 | 0.00001 |
